# Asparaginyl endopeptidase protects against podocyte injury in diabetic nephropathy through cleaving cofilin-1

**DOI:** 10.1038/s41419-022-04621-2

**Published:** 2022-02-25

**Authors:** Chuntao Lei, Mengran Li, Yang Qiu, Yaru Xie, Zhe Hao, Xingjie Yin, Zhentao Zhang, Hua Su, Linlin Yang, Jihong Lin, Hans-Peter Hammes, Chun Zhang

**Affiliations:** 1 Department of Nephrology, Union Hospital, Tongji Medical College, Huazhong University of Science and Technology, Wuhan, 430022 China; 2 Department of Neurology, Renmin Hospital of Wuhan University, Wuhan, 430060 China; 3 5th Medical Department, Medical Faculty Mannheim, Heidelberg University, Mannheim, Germany

**Keywords:** Cell death, Kidney diseases

## Abstract

Podocyte injury and loss are critical events in diabetic nephropathy (DN); however, the underlying molecular mechanisms remain unclear. Here, we demonstrate that asparaginyl endopeptidase (AEP) protects against podocyte injury through modulating the dynamics of the cytoskeleton. AEP was highly upregulated in diabetic glomeruli and hyperglycemic stimuli treated-podocytes; however, AEP gene knockout and its compound inhibitor treatment accelerated DN in streptozotocin-induced diabetic mice, whereas specific induction of AEP in glomerular cells attenuated podocyte injury and renal function deterioration. In vitro, elevated AEP was involved in actin cytoskeleton maintenance and anti-apoptosis effects. Mechanistically, we found that AEP directly cleaved the actin-binding protein cofilin-1 after the asparagine 138 (N138) site. The protein levels of endogenous cofilin-1 1-138 fragments were upregulated in diabetic podocytes, consistent with the changes in AEP levels. Importantly, we found that cofilin-1 1-138 fragments were remarkably unphosphorylated than full-length cofilin-1, indicating the enhanced cytoskeleton maintenance activity of cofilin-1 1-138. Then we validated cofilin-1 1-138 could rescue podocytes from cytoskeleton disarrangement and injury in diabetic conditions. Taken together, our data suggest a protective role of elevated AEP in podocyte injury during DN progression through cleaving cofilin-1 to maintain podocyte cytoskeleton dynamics and defend damage.

## Introduction

Diabetic nephropathy (DN) is a major microvascular complication of diabetes mellitus and the leading cause of chronic and end-stage renal disease (ESRD) worldwide [1]. Podocytes are critical components of the filtration barrier. Various insults under diabetic circumstances can trigger podocyte injury, resulting in the effacement of the foot process and apoptosis, as well as detachment from the basement membrane. These events contribute to the breakdown of the glomerular filtration barrier, thereby resulting in albuminuria [2]. During the past decade, a reduction in the density or number of podocytes has been considered an early and critical event in DN development [3, 4]. However, therapeutic options to prevent or reduce podocyte loss in glomerular diseases, including DN, are currently lacking [5, 6]. A better understanding of the mechanisms underlying podocyte loss is urgently needed to identify potential therapeutic targets.

Mammalian asparaginyl endopeptidase (AEP), also known as legumain, is a lysosomal cysteine protease that specifically cleaves its substrates after aspartyl bonds. AEP activation is autocatalytic and requires the sequential removal of C-terminal and N-terminal propeptides in an optimal acidic environment [7, 8]. Although AEP is mainly localized in the acidic endolysosomal system, activated AEP has also been demonstrated to remain active and functional in the cytoplasm, membrane, and extracellular areas [9, 10]. The overexpressed AEP is implicated in numerous human diseases, including immune disorders, cancer, and neurological diseases [11, 12]. Physiologically, AEP is most abundant in the kidney proximal tubule and is necessary for normal lysosomal protein degradation in proximal tubules [13]. Moreover, AEP exerts an anti-fibrotic effect through the degradation of matrix fibronectin during unilateral ureteral obstruction-induced fibrotic lesions in animal models [14, 15]. However, the role of AEP has not been explored in glomerular diseases, including DN. Therefore, in this study, we examined the role and related mechanism of AEP in the pathogenesis of DN.

Here, our results show a significant increase of AEP expression in podocytes under hyperglycemia and demonstrate its reno-protective role in diabetic kidney podocyte injury. Mechanistically, we show that AEP cleaved cofilin-1, an actin-binding protein, to promote actin dynamics to resist damage. Our findings provide new insights into the pivotal role of AEP in maintaining normal podocyte function during physiology and suggest a reno-protective role of the increased AEP expression in the diabetic kidney.

## Results

### AEP was expressed in normal kidneys and glomerular cells

Previous studies reported that AEP is quite restricted in proximal tubules, is weakly observed at the distal tubules, and is barely detected in the glomeruli and medulla of mice according to immunofluorescent assay [16]. To confirm the AEP expression profile in normal kidney glomeruli, we performed western blot analysis of different compartments of rat and mouse kidneys and conducted immunostaining of specimens from normal adult humans, rats, and mice. Our results showed that AEP was expressed at lower levels in the glomeruli and medulla than in cortical tubules, and was weakly expressed in papilla (Fig. S1A, B). Moreover, AEP was widely expressed in various cultured glomerular cell lines, including human podocytes, murine podocytes, murine parietal epithelial cells, rat glomerular cells, and rat glomerular endothelial cells (Fig. S1C).

### AEP was increased in diabetic glomeruli and podocytes in response to stimuli

**Fig. 1:**
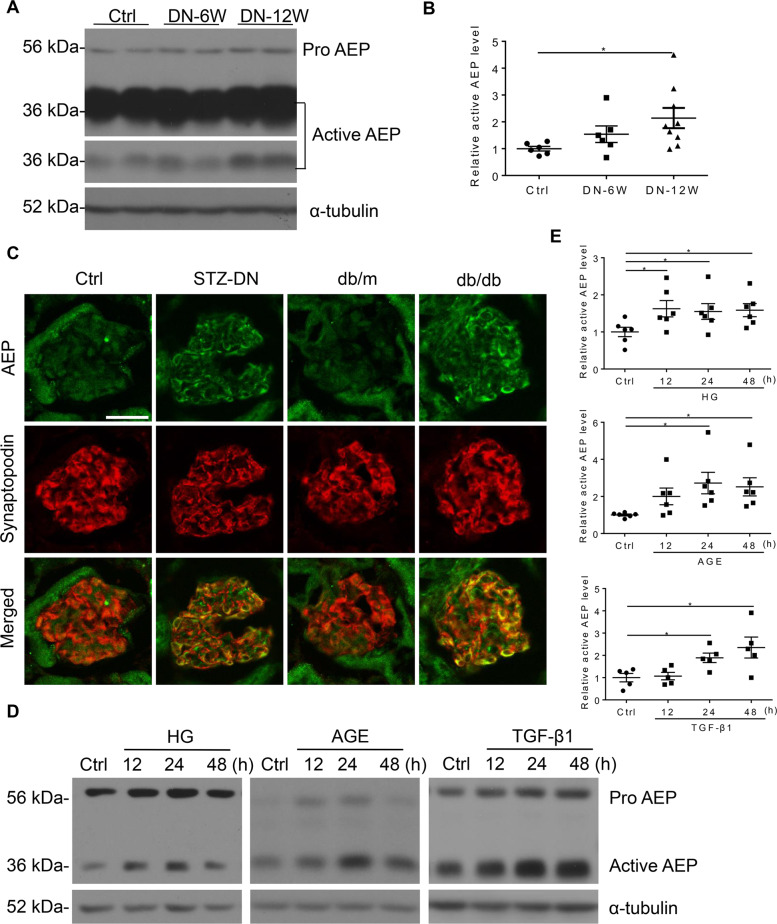
Asparaginyl endopeptidase (AEP) was upregulated in podocytes under diabetic conditions in vivo and in vitro. Representative western blot (**A**) and summarized data (**B**) showing AEP protein levels in the kidney cortex of control (Ctrl) and streptozotocin (STZ)-induced diabetic nephropathy (DN). *N* = 6–9. **C** AEP (green) colocalization with Synaptopodin (red) in the glomeruli of control mice and DN mice, db/m mice, and db/db mice. Scale bar: 25 μm. Representative western blot (**D**) and summarized data (**E**) showing AEP protein levels in human podocytes (HPCs) treated with high glucose (HG) (final concentration 30 mmol/L), and AGE (100 μg/mL), TGF-β1 (5 ng/mL). *N* = 5–6. *P < 0.05, data were represented as mean ± SEM.

Next, we examined the expression level of AEP, including the enzymatically inactive premature protein (56 kDa) and the fully mature active form (36 kDa), in DN animal kidney cortex tissues and in vitro hyperglycemia condition-stimulated podocytes. Western blot analysis showed that the protein levels of the two forms of AEP were increased in the streptozotocin (STZ)-induced diabetic cortex compared with the nondiabetic group (Fig. 1A), especially the active form as we analyzed and presented in Fig. 1B. By immunofluorescence assay, we found that AEP was significantly elevated in the glomerular area, and the increased AEP expression partially colocalized with the podocyte marker protein Synaptopodin (Fig. 1C). In vitro, we treated the cultured podocytes with common detrimental factors in diabetics, including high glucose (HG), advanced glycation endproducts (AGEs), or transforming growth factor-β1 (TGF-β1). We then detected the change in AEP expression by western blotting. The results revealed that multiple diabetic stimuli significantly increased the level of AEP expression in podocytes (Fig. 1D, E). These data suggest that increased AEP expression may contribute to glomerular damage, especially podocyte injury under diabetic conditions.

### AEP knockout exhibited more severe podocyte damage and glomerulopathy in diabetic mice

**Fig. 2:**
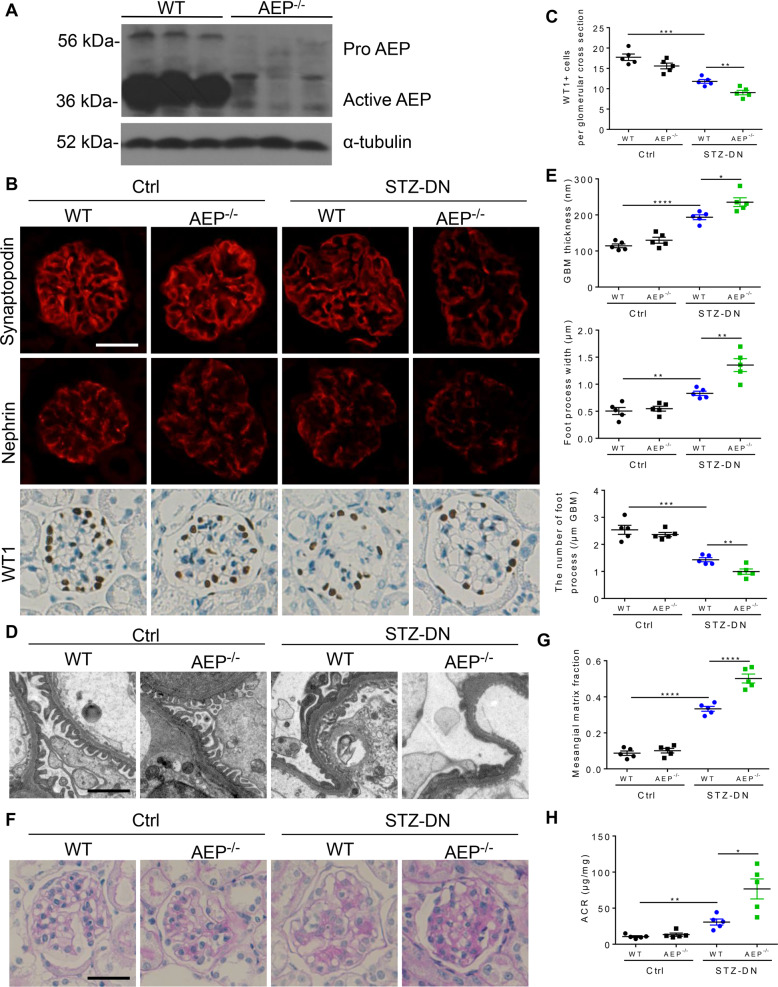
Diabetic glomerulopathy was aggravated in asparaginyl endopeptidase (AEP) knockout mice. **A** Western blot of AEP levels in wild-type (WT) and knockout mice (AEP^−/−^). *N* = 5. **B** Representative images for Synaptopodin, Nephrin, and WT1 in the control and STZ-induced glomeruli. Scale bar: 25 μm. **C** Quantitative analyses of the number of WT1-positive cells. *N* = 5. **D** Representative transmission electron microscopy (TEM) images showing morphological changes in the podocyte foot process in different groups of mice. Scale bar: 2 μm. *N* = 3. **E** Indices for glomerular filtration barrier integrity, including glomerular basement membrane (GBM) thickness, foot process width, and the number of foot processes/μm GBM. **F** Periodic acid-Schiff (PAS) staining showing glomerular morphological changes. Scale bar: 25 μm. **G** Quantitative analyses of the percentage of mesangial matrix area. *N* = 5. **H** Urinary albumin to creatinine ratio (ACR) in different groups of mice. *N* = 5. ****P < 0.0001, ***P < 0.001, **P < 0.01, *P < 0.05, data were represented as mean ± SEM.

To evaluate the role of AEP in the progression of DN, we validated the effect of AEP knockout on glomerulopathy, especially podocyte injury in the STZ-induced model. As described in the methods section, diabetes was induced in AEP knockout and control wild-type mice aged 7–8 weeks, and the mice were observed for 12 weeks. The blood glucose levels were comparable between STZ-injected knockout and wild-type mice, and the mice displayed similar extents of weight loss and kidney hypertrophy (Fig. S2). As shown in Fig. 2A, nearly no AEP expression was observed in knockout mice. Notably, greater podocyte injury was observed in the AEP knockout mice. Immunostaining revealed the decreased expression of podocyte marker proteins (Synaptopodin, Nephrin, and WT1) in diabetic AEP knockout glomeruli as compared with that in the wild-type group (Fig. 2B). In addition, increased podocyte loss was observed in diabetic AEP knockout kidneys, as demonstrated by observing WT1-positive cells (Fig. 2C). Electron microscopy confirmed the occurrence of exacerbated podocyte foot process effacement and glomerular basement membrane (GBM) thickening in the diabetic AEP knockout mice (Fig. 2D, E).

Consistent with increased podocyte injury and loss, histologic examination revealed significant changes in diabetic AEP knockout mice. Periodic acid-Schiff (PAS) staining of kidneys and semi-quantification of mesangial changes showed that significantly more prominent expansion of mesangial matrix occurred in AEP knockout animals (Fig. 2F, G). Moreover, when urine albumin to creatinine ratios (ACR) were assessed at 12 weeks after STZ injection, we observed that albuminuria was significantly increased in diabetic AEP knockout mice compared to that in wild-type mice (Fig. 2H).

### Administration of AEP inhibitor exacerbated STZ-induced diabetic glomerular lesions

Compound 11 is a highly specific small molecular inhibitor of AEP that interacts with both the active and allosteric sites of AEP, as described in a previous study [17]. We administered Compound 11 to mice after successful diabetes establishment and continued treatment for 14 weeks. The blood glucose levels, body weight, and kidney hypertrophy were comparable between the diabetic mice with or without Compound 11 treatment (Fig. S3). Immunostaining showed decreased podocyte expression of Synaptopodin and Nephrin, and a reduction of WT1-positive cells in diabetic kidneys chronically treated with Compound 11 (Fig. S4A, B). Electron microscopy also suggested exacerbated podocyte foot process effacement and GBM thickening in the Compound 11-treated diabetic mice (Fig. S4C, D). In addition, Compound 11 treatment accelerated matrix deposition and albuminuria in STZ-induced diabetic glomerular lesions (Fig. S4E–G). Altogether, inhibition of the protease activity of AEP aggravated podocyte injury and renal damage in the diabetic model. Consistent with the results from AEP knockout mice, our findings suggested a protective effect of AEP against diabetic podocyte injury and renal function deterioration in vivo.

### Induction of AEP expression in glomeruli attenuated podocyte injury in diabetic kidneys

**Fig. 3:**
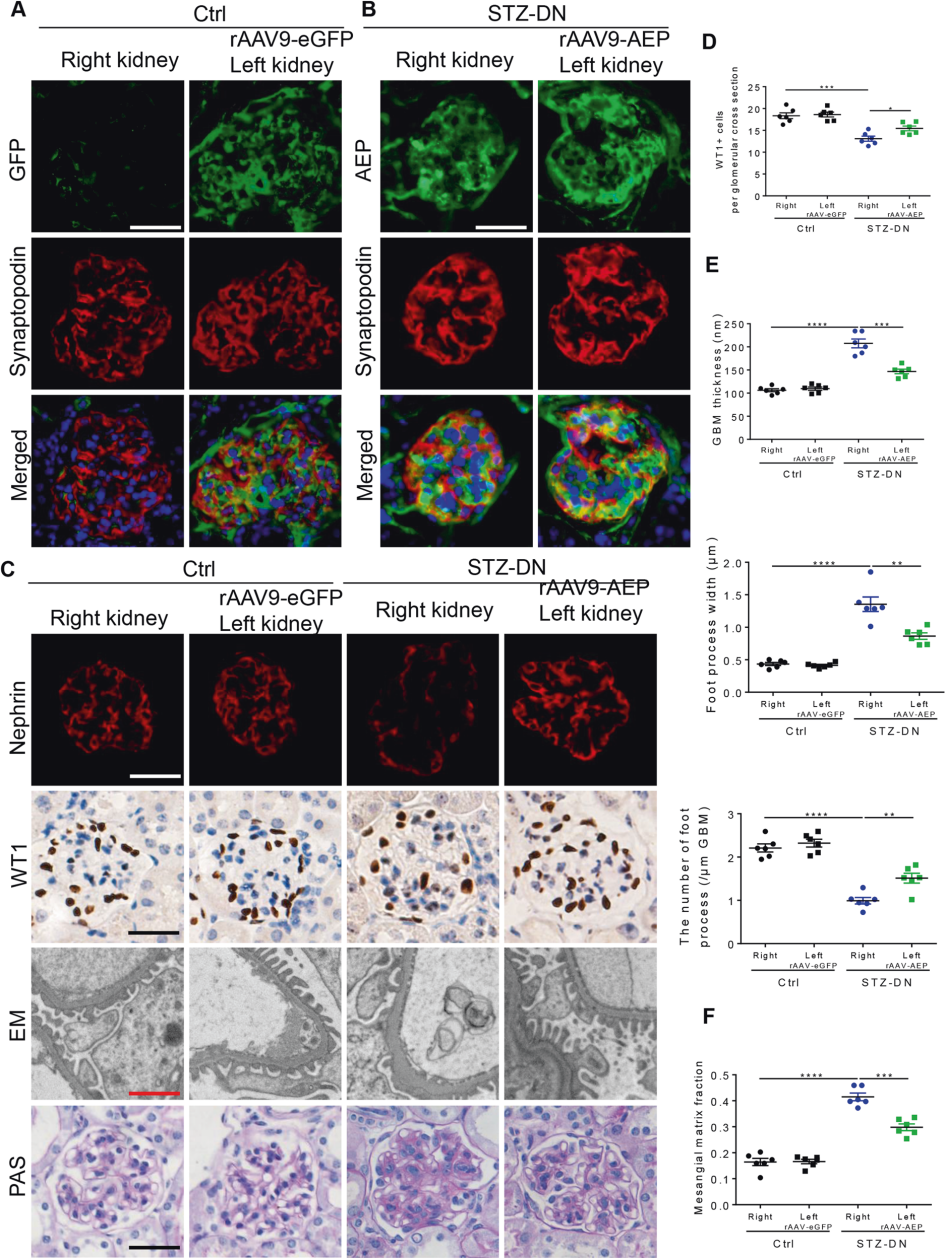
Induction of asparaginyl endopeptidase (AEP) expression in podocytes by intrarenal lentiviral gene delivery ameliorated podocyte injury and renal damage in streptozotocin (STZ)-induced diabetic nephropathy (DN). **A** Representative images of immunofluorescence co-staining between eGFP and Synaptopodin are shown to compare the eGFP expression and localization between the right and left kidneys (rAAV9-eGFP injected). Scale bar: 25 μm. **B** Representative image of immunostaining for AEP and Synaptopodin shows extensive overlap in rAAV9-AEP injected kidney glomeruli. Scale bar: 25 μm. **C** Representative image showing Nephrin and WT1 immunostaining, podocyte foot process, and glomerular basement membrane (GBM) and glomerular morphological changes in different groups of mice. White and black scale bar: 25 μm; red scale bar: 2 μm. **D** Quantification of WT1-positive cells, *N* = 6. **E** Indices for glomerular filtration barrier integrity, including GBM thickness, foot process width, and the number of foot processes/μm GBM. **F** Quantitative analyses of the percentage of mesangial matrix area. *N* = 6. ****P < 0.0001, ***P < 0.001, **P < 0.01, *P < 0.05, data were represented as mean ± SEM.

We next determined whether transient increased AEP expression in glomerular and podocyte was sufficient to attenuate diabetic kidney injury in vivo. We induced AEP overexpression in glomerular and podocytes of the left kidney through renal vein injection of rAAV9 expressing AEP driving by the podocyte-specific promoter (NPHS1), as described in the methods section. After recovery from rAAV9 injection operation, the mice were administrated with STZ to induce diabetes as described previously. The animals were observed in the following 12 weeks. We confirmed the transfection by performing western blot and fluorescence staining of eGFP and AEP and observing the expression in the cortex and the colocalized signal with Synaptopodin in glomeruli at an indicated time during the experiments. The eGFP signal was observed from the 3rd week, and the final results showed a robust and continuous expression of eGFP in the kidney cortex and colocalized with Synaptopodin in the glomeruli of the left kidney (Fig. S5A and Fig. 3A), suggesting high infection efficacy in glomerular, especially in podocytes. As expected, AEP-expressing rAAV9-injected left kidney showed significantly enhanced AEP expression in glomeruli and podocytes compared with the contralateral right kidney in the diabetic mice (Fig. S5B and Fig. 3B). Importantly, podocyte AEP induction resulted in a significant improvement in podocyte injury and loss, podocyte foot process effacement, and GBM thickening as stained against Nephrin and WT1 (Fig. 3C, D) or observed by electron microscope (Fig. 3C, E). A remarkable reduction in mesangial expansion was also observed in the rAAV9-AEP-injected left kidneys as compared with the un-injected right kidneys of STZ-induced diabetic mice (Fig. 3C, F).

### AEP mediated podocyte cytoskeleton arrangement and anti-apoptotic effects in vitro

**Fig. 4:**
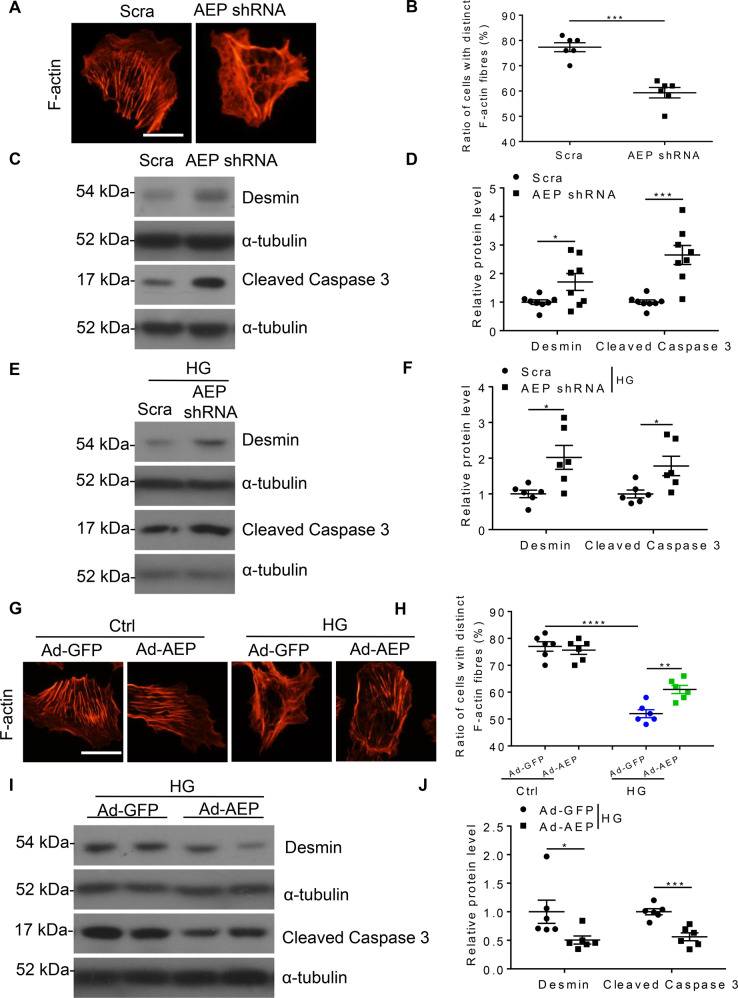
Asparaginyl endopeptidase (AEP) knockdown induced podocyte cytoskeleton disarrangement and cell injury and AEP overexpression reversed high glucose-induced podocyte injury. **A** Microscopic images of F-actin by rhodamine-phalloidin staining. Scale bar: 50 μm. **B** Summarized data from counting the cells with distinct, longitudinal F-actin fibers. Scoring was determined from 100 podocytes on each slide, *N* = 6. Representative western blot (**C**) and summarized data (**D**) showing Desmin and cleaved Caspase 3 protein levels in podocytes transfected with scrambled shRNA or AEP shRNA, *N* = 8. Representative western blot (**E**) and summarized data (**F**) showing Desmin and cleaved Caspase 3 protein levels in podocytes transfected with scrambled shRNA or AEP shRNA under high glucose (HG) stimulation conditions, *N* = 6. **G** Microscopic images of F-actin by rhodamine-phalloidin staining. Scale bar: 50 μm. **H** Summarized data from counting the cells with distinct, longitudinal F-actin fibers. Scoring was determined from 100 podocytes on each slide, *N* = 6. Representative western blot (**I**) and summarized data (**J**) showing Desmin and cleaved Caspase 3 protein levels in podocytes transfected with GFP adenovirus (Ad-GFP) or AEP adenovirus (Ad-AEP) under HG stimulation conditions. *N* = 6. ****P < 0.001, ***P < 0.001, **P < 0.01, *P < 0.05, data were represented as mean ± SEM.

To explore the role of AEP in vitro, we silenced AEP using shRNA lentivirus to determine whether reduced AEP expression affects podocyte injury under normal and diabetic conditions. The silencing effect was validated by western blot (Fig. S6A, B). First, we found that AEP depletion led to F-actin disarrangement (Fig. 4A, B) and an increase in Desmin (a marker of podocyte injury) and cleaved Caspase 3 (marker of cell apoptosis) expression in normal cultured podocytes (Fig. 4C, D). This suggested that silencing AEP expression induced podocyte cytoskeleton disarrangement and cell apoptosis. In addition, in podocytes incubated with HG, we found that transfection of AEP shRNA led to a further increase in Desmin and cleaved Caspase 3 expression, suggesting that reduced AEP expression exacerbated HG-induced podocyte injury (Fig. 4E, F). Similar podocyte damage was aggravated in TGF-β1-stimulated cells (Fig. S6C, D).

As the above in vivo and in vitro experiments showed the detrimental role of AEP knockdown and inhibition, we examined whether AEP overexpression can mitigate the HG- or TGF-β1-induced podocyte cytoskeleton disarrangement and cell injury. Podocytes were transfected with AEP adenovirus or control vector and a western blot assay was conducted to show the overexpression effect (Fig. S7A, B). To determine the effects of AEP on podocyte injury, we first examined cytoskeletal arrangement under HG-stimulated conditions with or without AEP overexpression. As shown in Fig. 4G, H, AEP upregulation reversed the HG-induced podocyte cytoskeleton disarrangement. In addition, Desmin and active Caspase 3 expression significantly decreased with AEP transfection (Fig. 4I, J). Similar F-actin and cell injury reversion was observed in TGF-β1-stimulated cells (Fig. S7C–F). These in vitro data suggest that AEP mediates the cytoprotective effect in podocytes under diabetic conditions.

### AEP directly cleaved cofilin-1

**Fig. 5:**
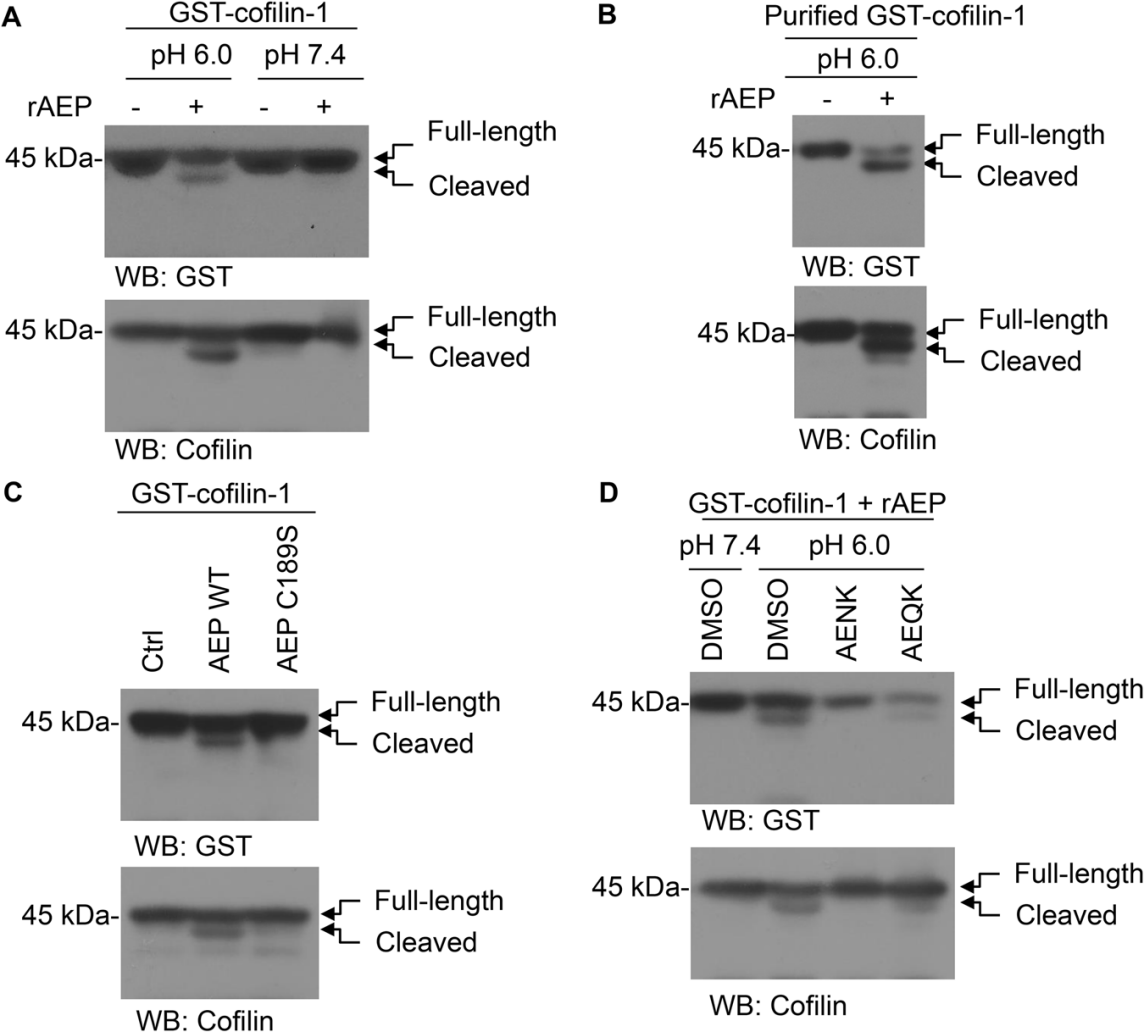
Asparaginyl endopeptidase (AEP) cleaved cofilin-1 in vitro. **A** Western blot showing the cleavage of GST-cofilin-1 after incubation with active recombinant AEP (rAEP) at pH 7.4 or pH 6.0. **B** Western blot showing the cleavage of purified GST-cofilin-1 recombinant protein by active recombinant AEP at pH 6.0. **C** Western blot showing the cleavage of cofilin-1 by wild-type (WT) and mutant AEP. **D** Western blot showing the effect of AENK and AEQK on cofilin-1 cleavage.

Cofilin-1 is an actin-binding protein that is essential for podocyte morphology during development and in response to podocyte injury [18]. To investigate whether cofilin-1 is a substrate of AEP, we incubated the whole lysates of cells transfected with GST-cofilin-1 plasmid with AEP under active (pH 6.0) or inactive (pH 7.4) conditions. Immunoblotting analysis revealed robust expression of the cofilin-1 cleavage fragment at pH 6.0, but not at pH 7.4 (Fig. 5A), a similar result was observed in the purified GST-cofilin-1 assay (Fig. 5B). Mutation of the key residue cysteine 189 (C189), which is essential for the cysteine proteinase activity of AEP, blunted its proteolytic activity against cofilin-1 (Fig. 5C). In addition, the selective AEP inhibitor peptide AENK suppressed cofilin-1 cleavage, while the inactive peptide AEQK had no effect (Fig. 5D). These data suggest that cofilin-1 is an enzymatic substrate of AEP.

### AEP cleaved cofilin-1 at N138 site in vitro and in vivo

**Fig. 6:**
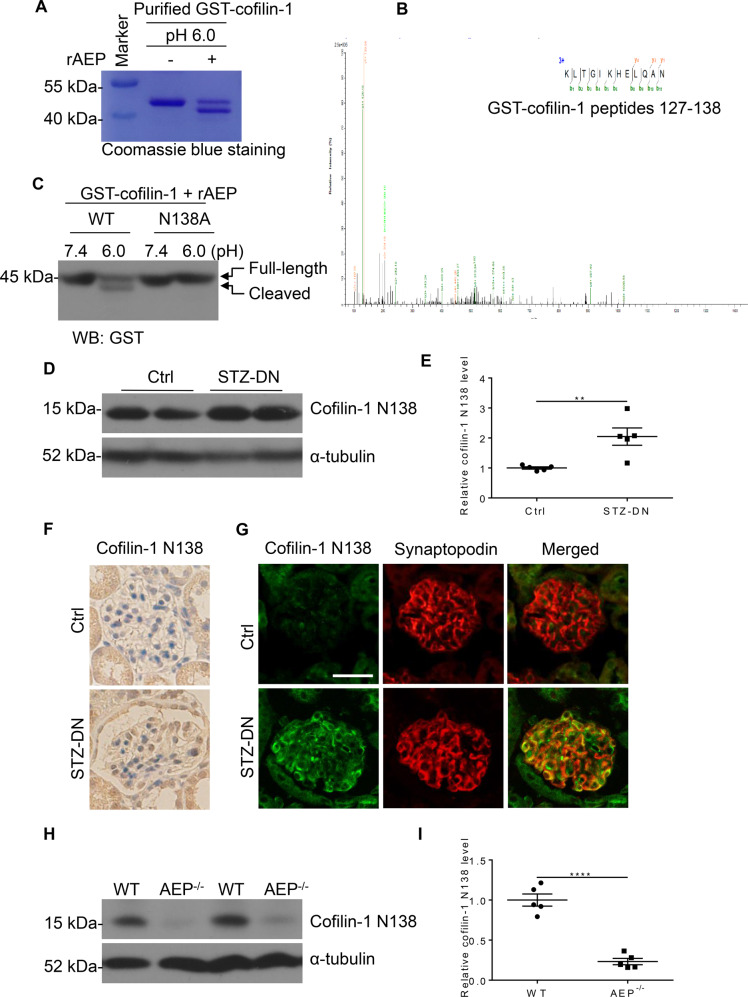
Asparaginyl endopeptidase (AEP) cleaved cofilin-1 at N138 in vitro and in vivo. **A** Cleavage of purified GST-cofilin-1 analyzed by Coomassie blue staining. **B** Mass spectrometry analysis of recombinant cofilin-1 fragmented by AEP. The detected MS/MS peptide spectra are listed. **C** Cofilin-1 cleavage was analyzed by western blot after GST-cofilin-1 wide-type, N138A, were incubated with active recombinant AEP. Western blot analysis (**D**) and summarized data (**E**) showing the levels of cofilin-1 N138 cleaved fragments in kidney cortex from control mice (Ctrl) and streptozotocin (STZ)-induced diabetic nephropathy (DN) mice (STZ-DN), *N* = 5. **F** Immunohistochemical staining of Ctrl and STZ-DN glomeruli. **G** Colocalization of cofilin-1 N138 fragment (green) with Synaptopodin (red), in the glomeruli of both Ctrl mice and STZ-DN mice. Scale bar 25 μm. Western blot analysis (**H**) and summarized data (**I**) showing the levels of cofilin-1 N138 cleaved fragments in kidney cortex from wild-type (WT) and AEP knockout (AEP^−/−^) mice. *N* = 5. ****P < 0.0001, **P < 0.01, data were represented as mean ± SEM.

To identify the potential AEP cleavage site on cofilin-1, we purified the cleaved recombinant protein products and analyzed them by mass spectrometry. We identified the main cleavage fragment ending at asparagine 138 (N138) (Fig. 6A, B). To further confirm this cleavage site, we mutated the asparagine 138 to alanine (N138A). The results showed that this mutation abolished the appearance of the cleavage product (Fig. 6C). To detect the presence of cofilin-1 fragments in DN kidney tissue, we developed an anti-cofilin-1 N138 antibody that specifically recognized the cleaved cofilin-1 1-138 band. Using western blot analysis and immunostaining with anti-cofilin-1 N138, we detected an increase in cofilin-1 1-138 fragments in diabetic kidney cortex and podocytes (Fig. 6D–G), suggesting that cofilin-1 cleavage correlated with the increase in AEP activity. Moreover, we detected cofilin-1 1-138 expression change in AEP knockout mice and the results showed cofilin-1 1-138 expressed corresponding to AEP level (Fig. 6H, I).

### Cofilin-1 1-138 fragments enhanced the regulation of actin dynamics to defend podocyte injury

**Fig. 7:**
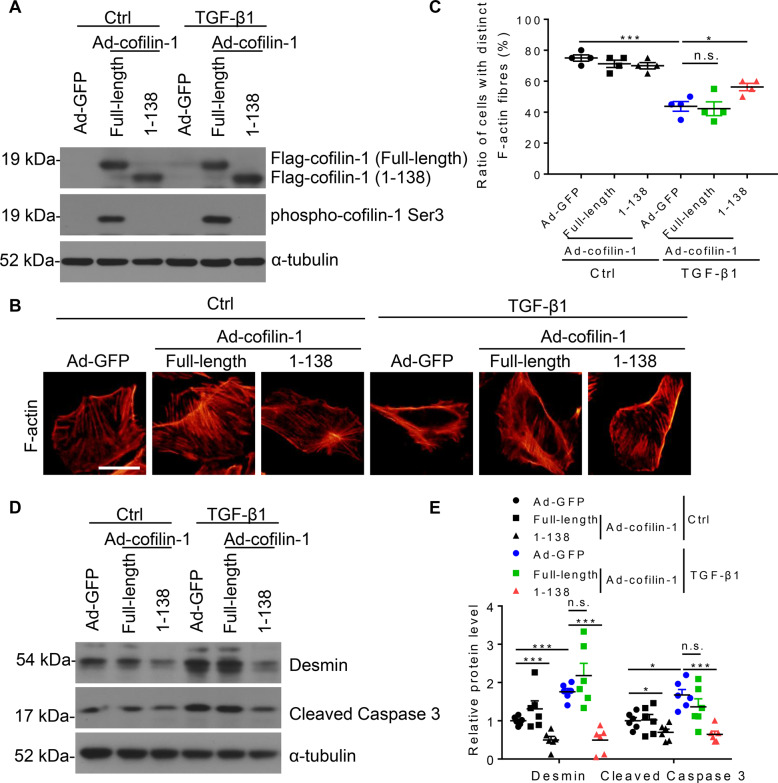
Decreased phosphorylation level of cofilin-1 1-138 fragments and its role in cultured podocytes. **A** Representative western blot showing the phosphorylation levels of cofilin-1 at serine 3 in podocytes transfected with full-length cofilin-1 or cofilin-1 1-138 adenovirus. **B** Microscopic images of F-actin by rhodamine-phalloidin staining. Scale bar: 50 μm. **C** Summarized data from counting the cells with distinct, longitudinal F-actin fibers. Scoring was determined from 100 podocytes on each slide. *N* = 4. Representative western blot (**D**) and summarized data (**E**) showing the protein levels of Desmin and cleaved Caspase 3 in podocytes transfected with green fluorescent protein (GFP) adenovirus, full-length cofilin-1, or cofilin-1 1-138 adenovirus. *N* = 6. ***P < 0.001, *P < 0.05, n.s. no significance, data were represented as mean ± SEM.

To assess whether AEP cleavage may affect the function of cofilin-1 in maintaining podocyte actin dynamics, we constructed full-length and fragment 1-138 cofilin-1 adenovirus vectors and transfected them into podocytes in vitro. As the phosphorylation status of serine 3 is the critical determinant of cofilin-1 activity, we first detected the phosphorylation levels of full-length cofilin-1 and the cofilin-1 1-138 fragment. Interestingly, the 1-138 fragment remained almost completely unphosphorylated than full-length cofilin-1 (Fig. 7A), indicating enhanced actin-binding and depolymerizing activity to maintain podocyte cytoskeleton to defend injury. As expected, the cofilin-1 1-138 fragment significantly attenuated TGF-β1-induced podocyte cytoskeleton disarrangement and decreased Desmin and active Caspase 3 expression in vitro, whereas full-length cofilin-1 did not (Fig. 7B–E).

**Fig. 8:**
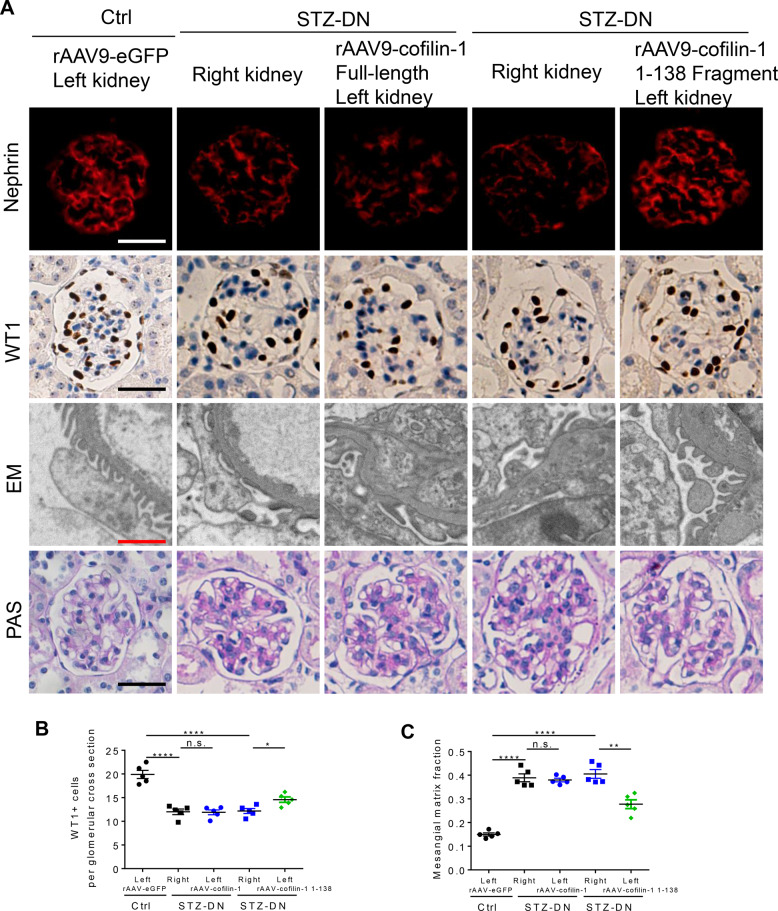
Induction of cofilin-1 1-138 fragment in podocytes improved podocyte injury and renal damage in streptozotocin (STZ)-induced diabetic nephropathy (DN). **A** Representative image showing Nephrin and WT1 immunostaining, podocyte foot process and glomerular basement membrane (GBM), and glomerular morphological changes in different groups of mice. White and black scale bar: 25 μm; red scale bar: 2 μm. **B** Quantification of WT1-positive cells. *N* = 5. **C** Quantitative analyses of the percentage of mesangial matrix area. *N* = 5. ****P < 0.0001, **P < 0.01, *P < 0.05, n.s. no significance, data were represented as mean ± SEM.

To confirm the role of the cofilin-1 1-138 fragment in vivo, we induced its expression by renal vein injection of rAAV9 expressing cofilin-1 or cofilin-1 1-138 driving by NPHS1. Compared with the corresponding right kidney of diabetic mice, the left kidney with cofilin-1 1-138 induction rescued podocyte injury and loss, and reduced mesangial expansion (Fig. 8). However, the cofilin-1 injection group did not show remarkable improvement in podocyte injury and histological lesions. Taken together, these in vitro and in vivo findings suggest that AEP mediates cytoprotection in podocytes during DN pathogenesis, partly through the production of the cofilin-1 1-138 fragment.

## Discussion

In the present study, we revealed for the first time an essential role of AEP in maintaining podocyte physiology function. Furthermore, the most important finding was the increased AEP expression elicited a protective role against hyperglycemia-associated cell injury in mice and cultured podocytes. Its potential mechanism is that AEP processes an actin-binding protein, cofilin-1, resulting in its enhanced actin-binding activity and the dynamic ability of the cytoskeleton to defend against damage. These findings enrich our understanding of the role of AEP in renal physiology and disease.

In the present study, we first demonstrated that AEP is indeed physiologically distributed lower signal in glomeruli than proximal tubules, and is widely expressed in glomerular cell lines, including human and murine podocytes. Furthermore, we demonstrated that AEP was upregulated in the podocyte regions of kidneys from STZ-induced type 1 and db/db type 2 diabetic mice, which are considered to be representative of the early stage of DN. In cultured podocytes, diabetic deleterious stimuli such as HG, AGEs, and TGF-β1 could induce high AEP expression. Meanwhile, we observed that AEP was also upregulated in mesangial areas of diabetic mice. Since podocyte injury and mesangial expansion are the two critical events during diabetic nephropathy [19, 20], remarkable changes in AEP expression may be implicated in DN progression.

In this study, we focus on the role of AEP in podocytes because we observed its prominent expression change in podocytes under diabetic conditions both in vivo and in vitro. Podocyte injury is considered an important early event and the strongest predictor of progressive DN, and it is meaningful to identify the relevant mechanisms responsible for podocyte injury. To explore the role of AEP in DN, we established an STZ-induced diabetic model in AEP knockout mice. Subsequently, podocyte injury, urinary albumin excretion, and the severity of glomerular injury were significantly aggravated in AEP knockout mice, suggesting that AEP protects against podocyte injury and renal damage. Although the previous study revealed that AEP knockout mice are prone to develop kidney abnormalities and lose normal renal function with aging, the AEP knockout mice in our study were observed within 20 weeks and the mice did not exhibit significant glomerular and tubular injury compared with age-matched wild-type mice at this stage, these data help to rule out the effect of AEP in the tubular compartment and suggest the reliability of the results in knockout mice.

In our previous study, we identified a nontoxic and selective compound inhibitor that specifically blocks AEP enzymatic activity [17]. Here, we treated STZ-induced diabetes C57BL/6 J mice with this compound and then evaluated the changes in kidney morphology and podocyte injury. Importantly, similar aggravated damage consistent with the AEP knockout effect were observed in the compound-administered mice. Combined with the consistent findings of AEP knockout and AEP inhibitor assay, we speculated that the elevated expression of AEP in diabetic glomerular might be a compensatory protective effect.

To validate whether increased AEP expression was sufficient to attenuate the DN in vivo, we established rAAV9 vector expression AEP driving by NPHS1 promoter, and then injected to mice through the renal vein to drive AEP overexpression specific in glomerular and podocytes, this method was considered to be optimal for glomerular-targeted gene delivery according to recent studies [21, 22]. After the mice recovered from virus injection surgery, we induced DN by STZ intraperitoneal injection, the resulting significant improvement of podocyte injury and loss of virus injected left kidney verified the therapeutic effect of AEP overexpression on podocytes. Moreover, the matrix deposition and expansion of the mesangium area also decreased remarkable, the related mechanisms maybe that AEP could directly degrade fibronectin similar as reported in the fibrotic animal model [14], and on the other hand, a previous study showed that AEP participated in the activation of matrix metalloproteinase 2 (MMP2) [23], a significant protein involved in matrix degrading, thus AEP may promote matrix degradation indirectly. These data confirm the upregulation of AEP induced by diabetic factors was protective against DN progression.

To assess whether AEP was directly responsible for podocyte damage, we knocked down and overexpressed AEP respectively in vitro to confirm its role in podocytes. We found that genetic inhibition of AEP at baseline induced cytoskeleton disarrangement and cell injury, and further aggravated the injury under diseased conditions. Conversely, overexpression of AEP alleviated podocyte’s impairment. These in vitro data suggested AEP protected podocytes against injury by the maintenance of actin cytoskeleton and anti-apoptosis activity, further studies are required to identify the related underlying mechanism.

AEP belongs to the cysteine protease family, contains a highly conserved motif with catalytic residues, and processes substrates with strict specificity. Growing evidence indicates that AEP is involved in a variety of diseases through processing substrate proteins. For example, AEP cleaves amyloid precursor protein and tau to play a critical role in neuronal cell death in Alzheimer’s disease [24, 25]. In the kidney, few AEP substrates associated with podocyte injury have been identified.

To identify proteins that may be processed by AEP, we performed proteomic analyses of AEP knockout and wild-type mice tissues (data not shown). Among differentially expressed proteins, we chose cofilin-1 for further investigation because cofilin-1 has previously been shown to be necessary for modulating actin filament dynamics, which are the structural backbone component of podocyte foot processes [26]. Cofilin-1 knockout animal models show dysfunction of the glomerular barrier and filtration, with foot process effacement and loss of secondary foot processes [18, 27]. Whether there is an exact correlation between AEP and cofilin-1 is unknown. In the present study, our data showed that AEP cleaved cofilin-1 after the N138 site to generate 1-138 fragments and cofilin-1 1-138 fragments were increased in diabetic kidneys, consistent with AEP upregulation level.

Cofilin-1 activity is inhibited by phosphorylation at serine 3, leading to a loss of actin-binding and severing activities and subsequently resulting in decreased cell actin filament elongation and remodeling ability [28, 29]. High glucose and TGF-β1 incubation leads to increased cofilin-1 phosphorylation and decreased cofilin-1 activation, resulting in podocyte actin cytoskeleton disruption [30]. Cyclosporine A protects podocytes from puromycin aminonucleoside (PAN)-induced injury via stabilization of cofilin-1 expression in its unphosphorylated state [31]. Stable knockdown of c-Maf-inducing protein prevents cofilin-1 phosphorylation and reorganization of the actin cytoskeleton in PAN-treated podocytes [32]. In our experiments, we found that the cofilin-1 1-138 fragment was not easily phosphorylated at amino acid serine 3, which might enhance cytoskeleton maintenance activity to protect podocytes from damage. In the following in vitro and in vivo experiments, we validated that cofilin-1 1-138 transfection engaged in podocyte cytoskeleton structure maintenance and had a protective effect against diabetic podocyte and glomerular injury, whereas the full-length form of cofilin-1 did not show protective effect because of being phosphorylated.

In summary, our study provides a better understanding of the essential biological role of AEP in kidney physiology and demonstrates for the first time that the increased expression of AEP is protective against podocyte injury in DN, through cleaving cofilin-1 to modulate the dynamic and anti-apoptotic actions of the cytoskeleton. AEP might be a potential therapeutic target for preventing podocyte injury and DKD progression.

## Materials and methods

### Human renal biopsy samples

The healthy human kidney samples were obtained from adult individuals who underwent benign tumor nephrectomy without any other renal diseases in Wuhan Union Hospital. Informed consent has been obtained from subjects.

### Animal study

C57BL/6 J male mice (7-week-old) and Sprague-Dawley (SD) male rats (7-week-old) were obtained from Charles River (Beijing, China). AEP knockout mice were provided by Dr. Keqiang Ye (Emory University School of Medicine, Atlanta, GA, USA). Animals were raised in a pathogen-free environment with a 12-h light and dark cycle and allowed free access to water and standard chow. The mice numbers of animal experiments were according to our previous study. Mice with poor physical condition before grouping and mice that died during the experiment were excluded. We used the random number method for random allocation and were blinded to allocate animals and assess the results during the experiments.

### Streptozotocin-induced DN in mice

Diabetes was induced by an intraperitoneal injection of streptozotocin (Sigma-Aldrich, Cat No. V900890, 150 mg/kg) as previously described [33]. Blood glucose level was measured 2 weeks later two times, and only the mice with glucose levels above 16.7 mmol/L were included in the following experiment. Blood glucose levels were monitored every 2 to 4 weeks.

### AEP inhibitor treatment in STZ-induced diabetic mice

Healthy and diabetic mice were divided into four groups, healthy control (receiving vehicle or Compound 11 treatment), diabetic mice (receiving vehicle or Compound 11 treatment). Compound 11 inhibitor (Matrix Scientific, Cat No. 033321) or vehicle group and were orally administered with 10 mg/kg body weight Compound 11 or vehicle after diabetes was established for 16 weeks. Urine samples, serum samples, and kidney tissues were collected.

### rAAV9 transduction of AEP and cofilin-1 1-138 fragment in mice

For induction of AEP and cofilin-1 expression in glomeruli, full-length AEP and cofilin-1 or the cofilin-1 1-138 fragment were inserted into an NPHS1 promoter (podocyte-specific in the kidney) to regulate the podocyte-specific expression of the enhanced green fluorescent protein (eGFP) vector [21]. Recombinant pseudotyped adeno-associated virus serotype 9 (rAVV9) was packaged, purified, and titrated by Vigene Biosciences (Shandong, China). rAAV9 (1 × 10^12^ copies) harboring either the target gene or the eGFP vector was injected through the left renal vein of the mice, as described previously [22, 34]. In brief, animals were given a general anesthetic, and the left kidney of the mouse was exposed using a flank incision. The renal vein was clamped using a microaneurysm clamp, and rAAV9 particles diluted in 100 μL saline was injected into the vein using a 31-gauge needle. The clamp was removed 15 min after injection, and the incision was sutured.

### Glomeruli isolation

To identify the target protein expression profile, mouse glomeruli were isolated as described previously [22]. In brief, mice were anesthetized and perfused with 8 × 10^7^ Dynabeads M-450 Tosylactivated (Invitrogen, Cat No. 14013) diluted in phosphate-buffered saline (PBS) through the heart. The kidneys were removed and the cortex, medulla, and papilla were separated by dissection. Cortex was minced into 1 mm^3^ piece and digested in collagenase (Sigma-Aldrich, Cat No. C6885) at 37 °C for 15 min. Then, the tissue was pressed through a 100 μm cell strainer, and the cell suspension was centrifuged for the pellet. The pellet was resuspended in PBS, and the glomeruli containing Dynabeads were gathered using a magnetic particle concentrator and washed at least three times with PBS. The supernatant enriched with proximal tubules was washed and collected for analysis.

Rat glomeruli were isolated as previously described [35]. Briefly, the renal cortexes of SD rats were minced and gently pressed through a series of sieves (200, 120, and 75 μM), and the glomeruli were then gathered on the last sieve.

### Urine albumin and creatinine measurement

Urine creatinine levels were measured using an auto-chemistry analyzer according to the creatinine assay kit. Urine albumin was detected using an ELISA kit (Abcam, Cambridge, Cat No. ab108792) according to the manufacturer’s instructions. The urine albumin excretion rate was expressed as the ratio of albumin to creatinine.

### Cell culture and treatments

A conditionally immortalized human podocyte cell line was cultured and maintained as described previously [36]. In brief, podocytes were cultured in Roswell Park Memorial Institute 1640 medium (RPMI 1640) supplemented with 10% fetal bovine serum, 100 U/mL penicillin, and 100 U/mL streptomycin at 33 °C for proliferation. Upon reaching appropriate confluence, the cells were maintained at 37 °C for 10–14 days to induce differentiation. Differentiated podocytes were exposed to different stimuli for certain times to mimic the diabetic environment: HG (Sigma-Aldrich, Cat No. 49163, 30 mmol/L), AGE (Abcam Cambridge, Cat No. ab51995, 100 μg/mL), and TGF-β1 (PeproTech, Cat No. 100-21, 5 ng/mL).

HEK293T cells were cultured for plasmid transfection experiments in Dulbecco’s modified Eagle’s medium (DMEM) containing 10% fetal bovine serum. The cultured cells were regularly screened for mycoplasma contamination by our research group.

### RNA interference and gene overexpression of podocyte

A lentivirus vector harboring a short-hairpin RNA (shRNA) sequence targeting AEP was synthesized by Jikai Gene (Shanghai, China), and scrambled shRNA was used as a control. Adenovirus vectors containing the DNA target sequence for AEP, full-length cofilin-1, and cofilin-1 1-138 fragments were obtained from Vigene Biosciences (Shandong, China). Podocytes were transiently transfected with lentivirus or adenovirus, according to the manufacturer’s instructions, to interfere with AEP expression or induce AEP, cofilin-1, and cofilin-1 1-138 overexpression.

### In vitro cofilin-1 cleavage assay

To assess the cleavage of cofilin-1 by AEP in vitro, HEK293T cells were transfected with GST-tagged cofilin-1 plasmids using Lipofectamine 2000 (Invitrogen, Cat No. 11668019). Forty-eight hours after transfection, the cells were collected, washed with PBS, lysed in lysis buffer (Beyotime Biotechnology, Cat No. P0013), and centrifuged for 15 min at 12,000 rpm at 4 °C to obtain the supernatant. Then, the supernatant was incubated with recombinant AEP in the indicated pH buffer at 37 °C for 15 min. To measure the cleavage of purified cofilin-1 by AEP, GST-tagged protein was purified with glutathione beads (Invitrogen, Cat No. G2879) according to the manufacturer’s instructions. The purified cofilin-1 was incubated with recombinant AEP. In addition, cells were co-transfected GST-cofilin-1 with wild-type AEP or mutant AEP (C189S) with abolished cysteine protease activity, and the supernatant was incubated at pH 6.0. To test the effect of AEP peptide inhibitor on the cleavage of cofilin-1 by AEP, the AENK peptide inhibitor and inactive control AEQK were co-incubated with recombinant AEP and GST-cofilin-1 supernatant at pH 6.0. The samples were then boiled in 1× SDS loading buffer and analyzed using immunoblotting.

To identify the cofilin-1 cleavage site by AEP, the recombinant AEP incubated purified cofilin-1 samples were subjected to SDS-PAGE and then stained using Coomassie blue buffer (Beyotime Biotechnology, Cat No. P0017), the target gel was further analyzed by mass spectrometry. In addition, the indicated asparagine site of the cofilin-1 plasmid was mutated to alanine to validate the cleavage.

### Generation of antibodies that specifically recognize the AEP-generated cofilin-1 fragment (anti-cofilin-1 N138)

The rabbits were immunized with the peptide Ac-TGIKHELQAN-OH, which includes the nine amino acids in cofilin-1 preceding the AEP cleavage site at N138. The antiserum was pooled and the titers against the immunizing peptide were determined by ELISA. The maximal dilution giving a positive response using the chromogenic substrate for HRP was 1:30 000. The immunoactivity of the antiserum was further confirmed by western blotting and immunohistochemistry.

### Western blotting

The total protein lysates of cultured cells and kidney tissues were prepared and western blot analysis were performed as described previously [36]. The following primary antibodies were used in this study: AEP (R&D, Cat No. AF2199, 1:1 000); Desmin (Bioworld Technology, Cat No. BS1712, 1:1 000); Caspase 3 (Protein Tech Group, Cat No. 19677-1-AP, 1:1 000); GST (Protein Tech Group, Cat No. 10000-0-AP, 1:3 000); Cofilin (Cell Signaling Technology, Cat No. 5175, 1:1 000); Phospho-Cofilin (Ser3) (Cell Signaling Technology, Cat No. 3313, 1:1 000); Cofilin-1 N138 (1:500); GFP (Protein Tech Group, Cat No. 50430-2-AP, 1:2 000), and α-tubulin (Protein Tech Group, Cat No. 66031-1-Ig, 1:3 000).

### Immunohistochemistry

Kidney tissues were fixed using 4% paraformaldehyde and embedded in paraffin. Four-micrometer sections were deparaffinized and rehydrated for immunohistochemical staining using the standard biotin-streptavidin-peroxidase method as described previously [37]. Briefly, endogenous peroxidase was blocked with 5% bovine serum albumin for 30 min at room temperature, and then the sections were incubated with the primary antibody for AEP (Santa Cruz, Cat No. sc-133234, 1:50), WT1 (Abcam Cambridge, Cat No. ab89901, 1:100) and Cofilin-1 N138 (1:50) overnight at 4 °C. Then, sections were washed in PBS and incubated with secondary antibody and HRP-labeled streptavidin, each for 20 min. Finally, peroxidase activity was visualized by diaminobenzidine, and sections were counterstained with hematoxylin before the inspection. For quantification of WT1-positive cells, at least 20 glomeruli sections per slide were counted.

### Immunofluorescence staining

Kidney sections of experimental mice were prepared and indirect immunofluorescence staining was performed as described previously [38, 39]. The following primary antibodies were used: anti-AEP (Santa Cruz, Cat No. sc-133234, 1:50), anti-Synaptopodin (Synaptic Systems, Cat No. 163004, 1:200), anti-Nephrin (R&D, Cat No. AF3159, 1:100), and anti-cofilin-1 N138 (1:50). The secondary antibodies: Alexa Fluor 488 IgG (Invitrogen, Cat No. S11223, 1:200) and Alexa Fluor 594 IgG (Invitrogen, Cat No. A10438, 1:200). Nuclear was counterstained with Hoechst (Beyotime Biotechnology, Cat No. C1011).

### F-actin staining

To assess podocyte cytoskeleton arrangement, podocytes were cultured on glass coverslips and subjected to indicated treatments. Then, the cells were fixed using 4% paraformaldehyde solution for 10 min. and incubated with rhodamine-phalloidin (Sigma-Aldrich, Cat No. P1951) in PBS containing 10% donkey serum and 0.3% Triton X-100 for 30 min before observation. One hundred cells were counted to calculate the ratio of cells retaining distinct F-actin fibers in different groups.

### Transmission electron microscopy (TEM)

Electron microscopic sample handling and detection were performed using an electron microscopic core. TEM images were analyzed using Image J software (NIH, Bethesda, MD, USA), and the GBM thickness, foot process width, and the number of foot processes per micrometer of GBM were calculated as described earlier [40].

### Morphological examination

PAS staining of four-micrometer paraffin sections was used to examine the mesangial expansion in the glomeruli. Mesangial and glomerular cross-sectional areas were quantified using image-pro plus 6.0 software (Media Cybernetics, Rockville, MD, USA) and a minimum of 20 glomeruli per section were measured.

### Statistics

All experiments in this study were repeated at least three times and representative experiments are shown. The results are reported as the mean ± SEM. Normally distributed data were analyzed and graphed by GraphPad Prism software (GraphPad, San Diego, CA, USA). Student’s *t*-test was used to compare two experimental groups, and all tests were two-tailed, and *p* < 0.05 was considered statistically significant.

## Supplementary information

Supplemental figures

Western blot gel images

Reproducibility Checklist

## Data Availability

All data analyzed during this study are included in this published article and are available upon request by contacting the corresponding author.
