# Supplementary material for: Asparaginyl endopeptidase protects against podocyte injury in diabetic nephropathy through cleaving cofilin-1
Source: Cell Death Dis. 2022 Feb 25;13(2):184. doi: 10.1038/s41419-022-04621-2 (PMC8881581; doi:10.1038/s41419-022-04621-2)
Supplement: Supplementary file 1 — Supplemental figures [file 41419_2022_4621_MOESM1_ESM.pdf]

Supplemental figures and Figure legends

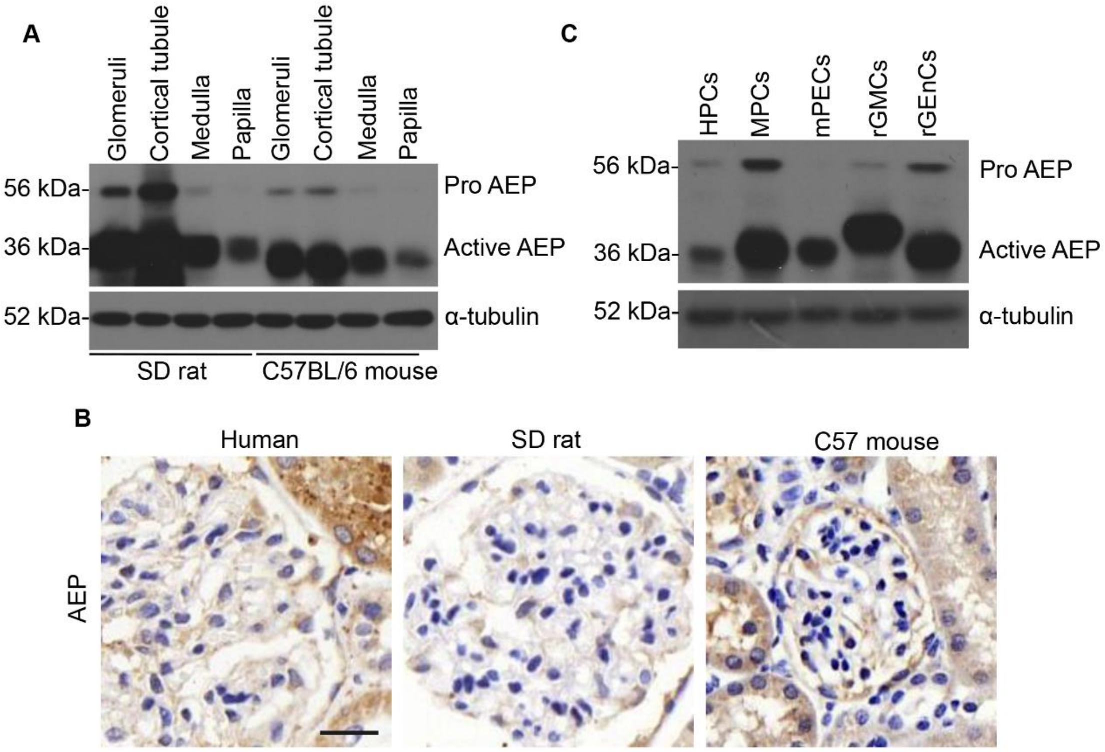

Figure S1. Expression profile of asparaginyl endopeptidase (AEP) in normal kidney tissues and glomerular cell lines. (A) Representative western blot showing relative AEP protein levels in glomeruli, cortical tubules, the medulla, and papilla tissues. (B) Immunohistochemical staining of AEP in human, rat, and mouse glomeruli. Scale bar: 25 μm. (C) Representative western blot showing relative AEP protein levels in cultured renal human podocytes (HPCs), mouse podocytes (MPCs), mouse parietal epithelial cell (mPECs), rat glomerular mesangial cells (rGMCs), and rat glomerular endothelial cells (rGEnCs). SD: Sprague-Dawley rat; C57: C57BL/6J mouse.

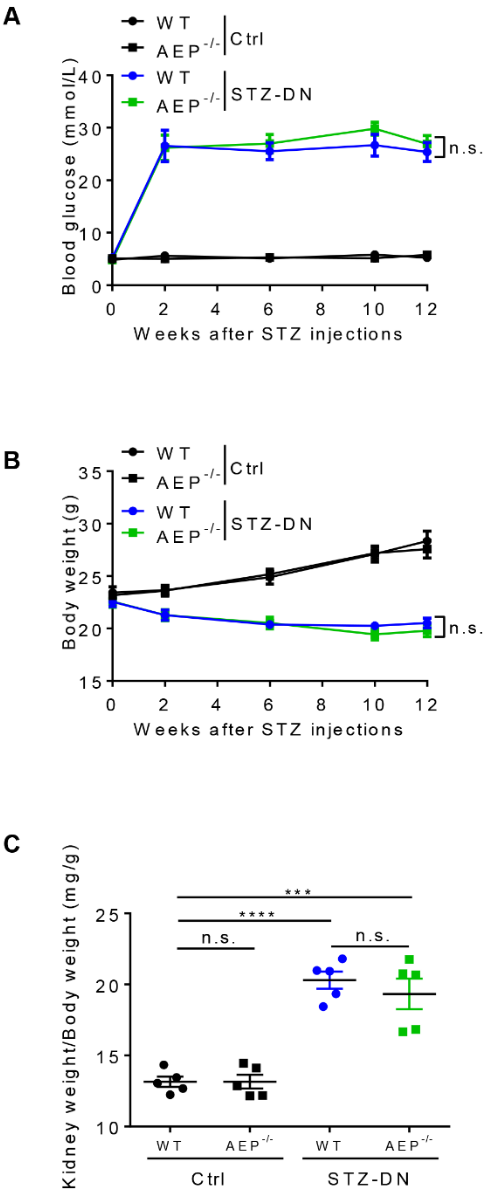

Figure S2. Physical parameters in wildtype and AEP knockout diabetic mice. Blood glucose (A) and body weight (B) monitoring of wildtype and AEP knockout mice after induction of diabetes. (C) Kidney-to-body weight ratio at the end of experiment. N = 5. ****$P < 0.0001$, n.s. no significance. Data are represented as mean ± SEM. Ctrl: control; STZ-DN: streptozotocin-induced diabetic nephropathy; WT: wildtype; AEP$^{-/-}$: AEP knockout.

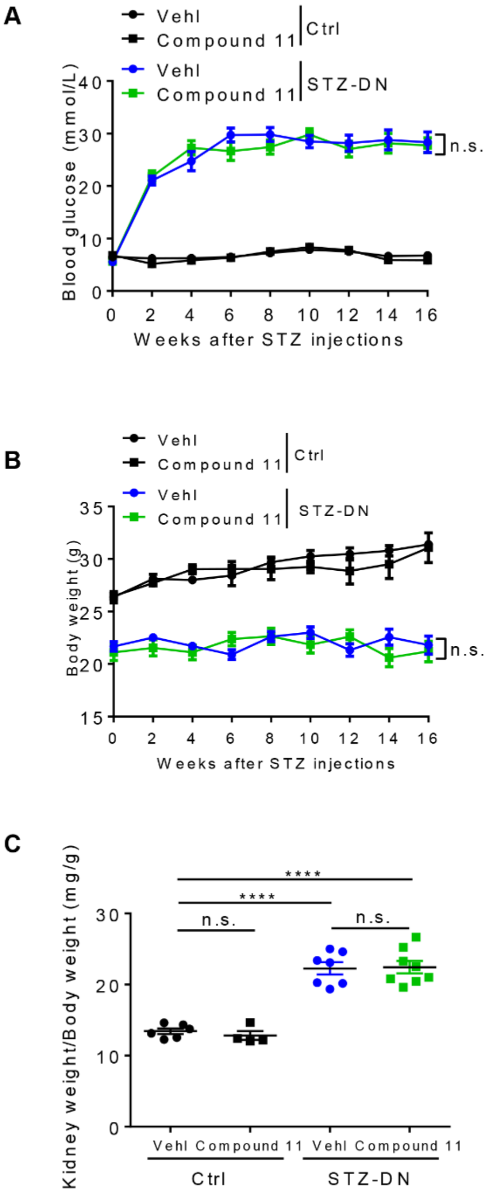

Figure S3. Physical parameters in diabetic mice treated with or without AEP inhibiting compound. Blood glucose (A) and body weight (B) monitoring in mice treated with vehicle or Compound 11 after induction of diabetes. (C) Kidney-to-body weight ratio at 16 weeks after STZ injection. N = 4-8. ****P < 0.0001, n.s. no significance. Data are represented as mean ± SEM. Ctrl: control; STZ-DN: streptozotocin-induced diabetic nephropathy; Vehl: vehicle.

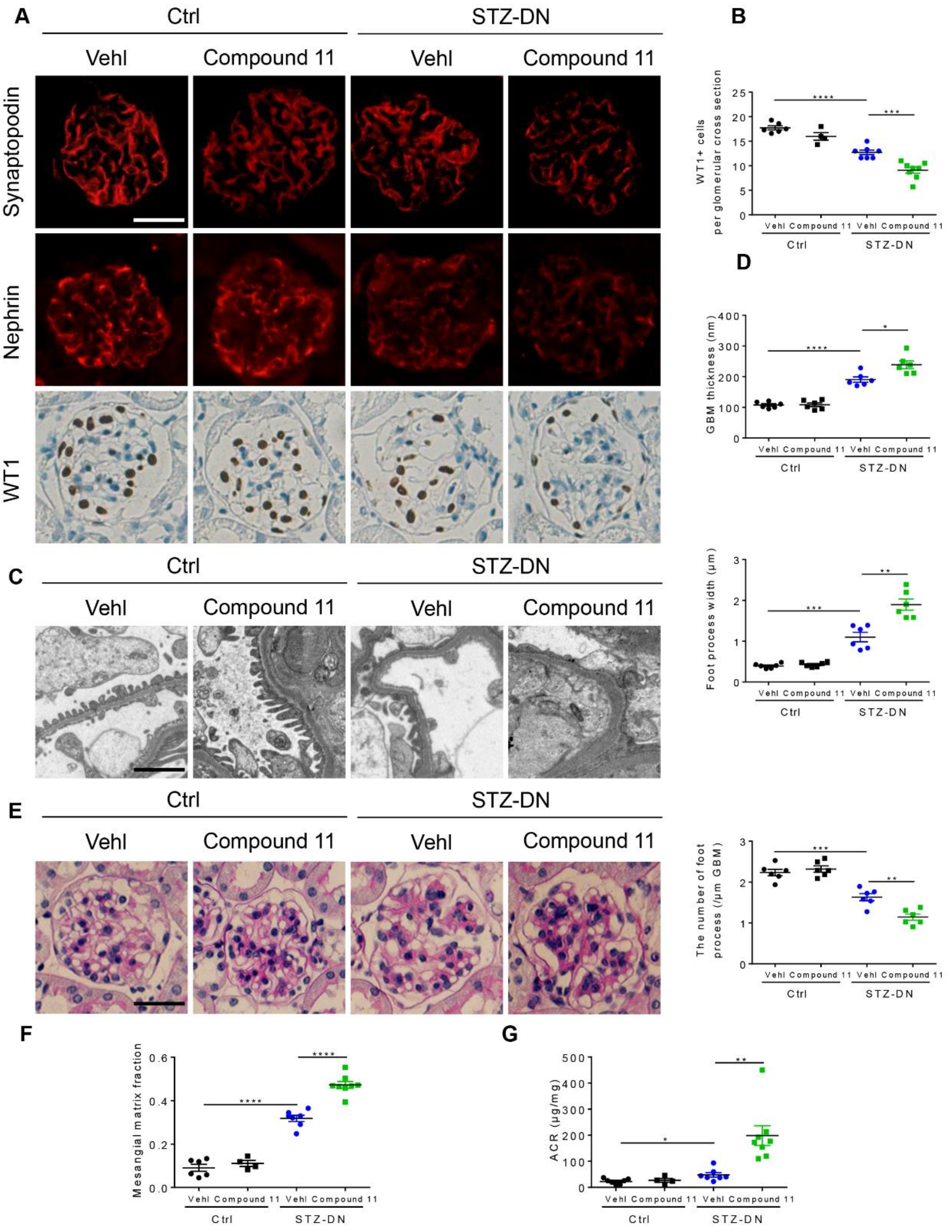

Figure S4. Asparaginyl endopeptidase (AEP) inhibitor exacerbated podocyte injury in diabetic nephropathy (DN). (A) Representative images for Synaptopodin, Nephrin, and WT1 in the control and STZ-induced glomeruli treated with or without Compound 11. Scale bar: 25 μm. (B) Quantitative analyses of the number of WT1-positive cells. (C) Representative transmission electron microscopy (TEM) images showing morphological changes in the podocyte foot processes in different groups of mice. Scale bar: 2 μm. (D) Indices for glomerular filtration barrier integrity, including glomerular basement membrane (GBM) thickness, foot process width and the number of foot processes/μm GBM. (E) Periodic acid-Schiff (PAS) staining showing

glomerular morphological changes. Scale bar: 25 μm. (F) Quantitative analyses of the percentage of mesangial matrix area. (G) Urinary albumin to creatinine ratio (ACR) in different groups of mice. N = 4–8. *****P* < 0.0001, ****P* < 0.001, ***P* < 0.01, **P* < 0.05. Data are represented as mean ± SEM. Ctrl: control; STZ-DN: streptozotocin-induced diabetic nephropathy; Vehl: vehicle.

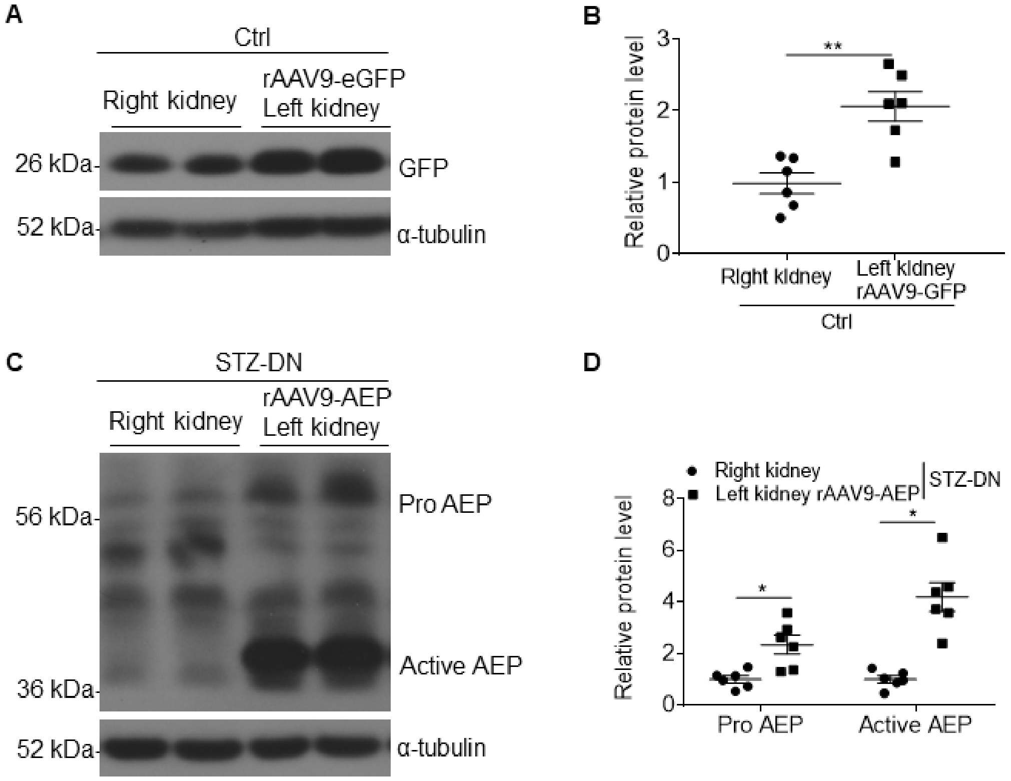

Figure S5. Transfection of AEP by renal intravenous rAVV9 injection. Representative western blot (A) and summarized data (B) showing GFP protein levels in the right and left kidney cortex from rAAV9 expressing eGFP left renal vein injection mice. N=6. ***P* < 0.01. Representative western blot (C) and summarized data (D) showing AEP levels in the right and left kidney cortex from rAAV9 expressing AEP left renal vein injection diabetic mice. N=6. **P* < 0.05. Data are represented as mean ± SEM. Ctrl: control; STZ-DN: streptozotocin-induced diabetic nephropathy.

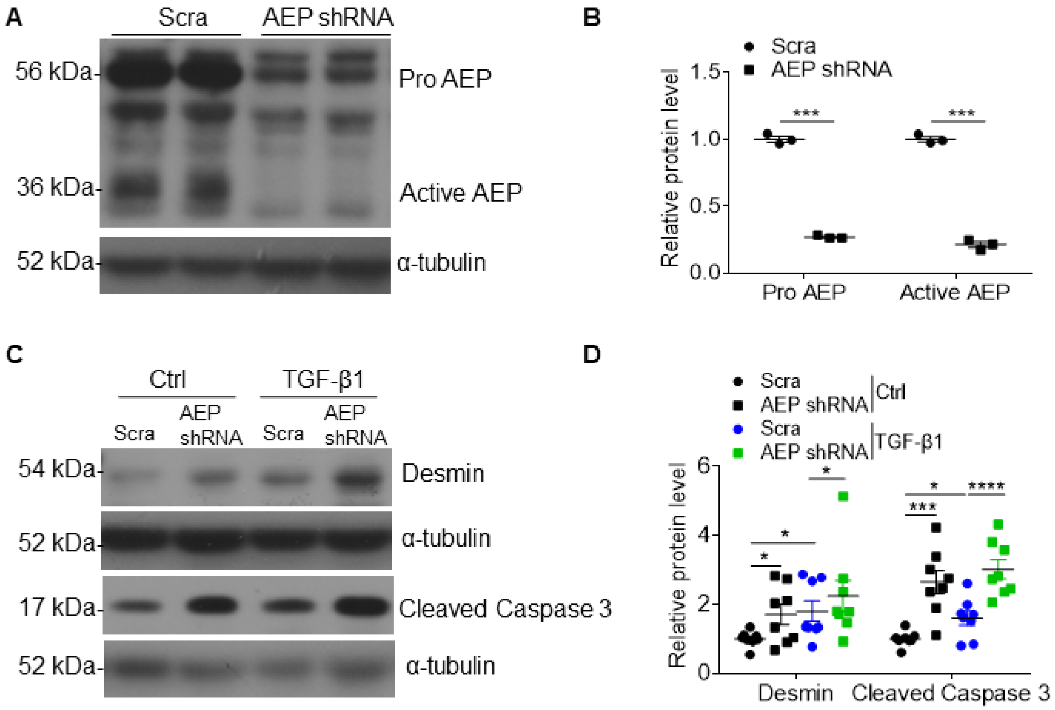

Figure S6. Asparaginyl endopeptidase (AEP) knockdown aggravated cell injury and apoptosis in TGF-β1 stimulated podocytes. Representative western blot (A) and summarized data (B) showing AEP protein levels in podocytes transfected with scrambled shRNA (Scra) or AEP shRNA. Representative western blot (C) and summarized data (D) showing Desmin and Cleaved Caspase 3 protein levels change in podocytes transfected with scrambled shRNA (Scra) or AEP shRNA under TGF-β1 stimulated condition. N = 3–8. *****P* < 0.0001, ****P* < 0.001, **P* < 0.05. Data are represented as mean ± SEM. Scra: scramble shRNA; Ctrl: control.

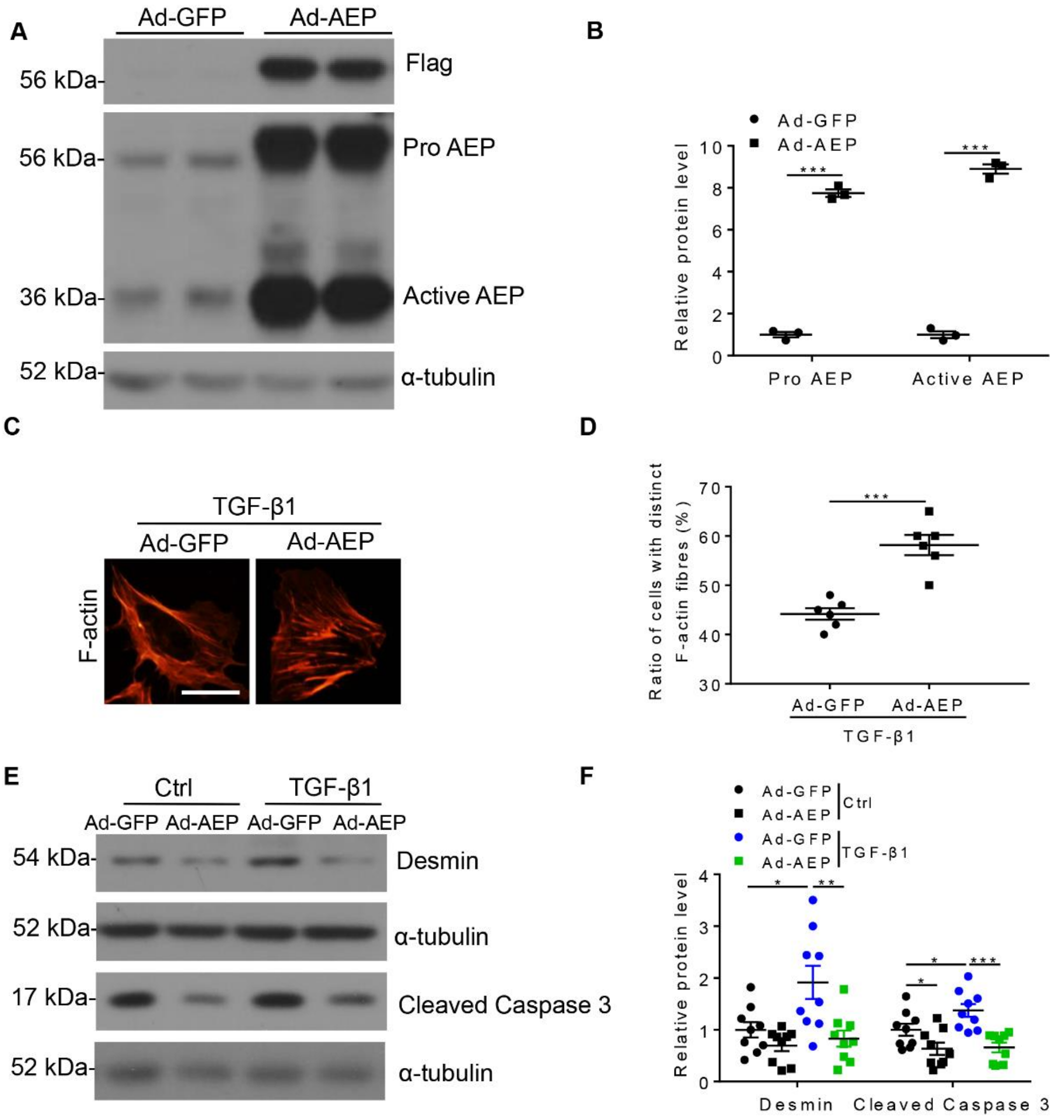

Figure S7. AEP overexpression reversed TGF-β1-induced cytoskeleton disarrangement and podocyte injury. Representative western blot (A) and summarized data (B) showing AEP protein levels in podocytes transfected with green fluorescent protein (GFP) or AEP adenovirus. (C) Microscopic images of F-actin by rhodamine-phalloidin staining. Scale bar: 50 μm. (D) Summarized data from counting the cells with distinct, longitudinal F-actin fibres. Representative western blot (E) and summarized data (F) showing Desmin and cleaved Caspase 3 protein levels in podocytes transfected with Ad-GFP or Ad-AEP exposed to TGF-β1. Scoring was determined from 100 podocytes on each slide. N = 3–9. ***$P < 0.001$, **$P < 0.01$, *$P < 0.05$. Data are represented as mean ± SEM. Ad-GFP: GFP adenovirus; Ad-AEP: AEP adenovirus; Ctrl: control.
