# Supplementary material for: Asparaginyl endopeptidase protects against podocyte injury in diabetic nephropathy through cleaving cofilin-1
Source: Cell Death Dis. 2022 Feb 25;13(2):184. doi: 10.1038/s41419-022-04621-2 (PMC8881581; doi:10.1038/s41419-022-04621-2)

Figure 1-A

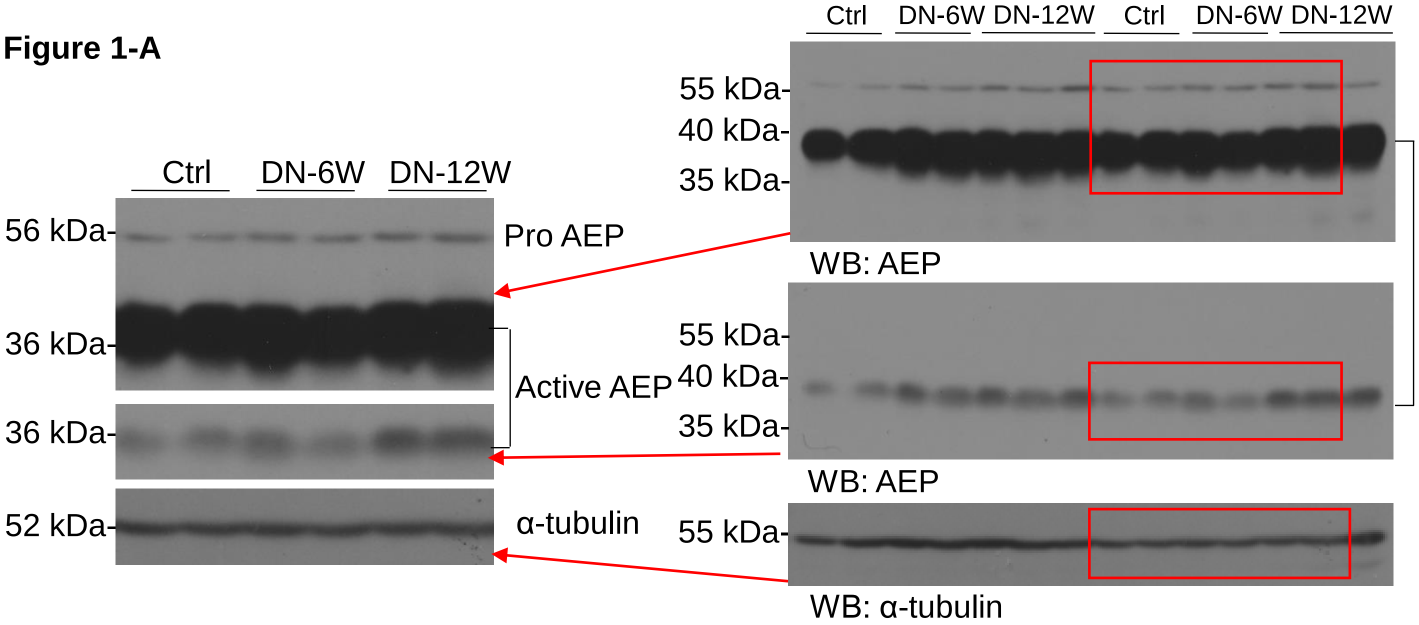

Figure 1-D

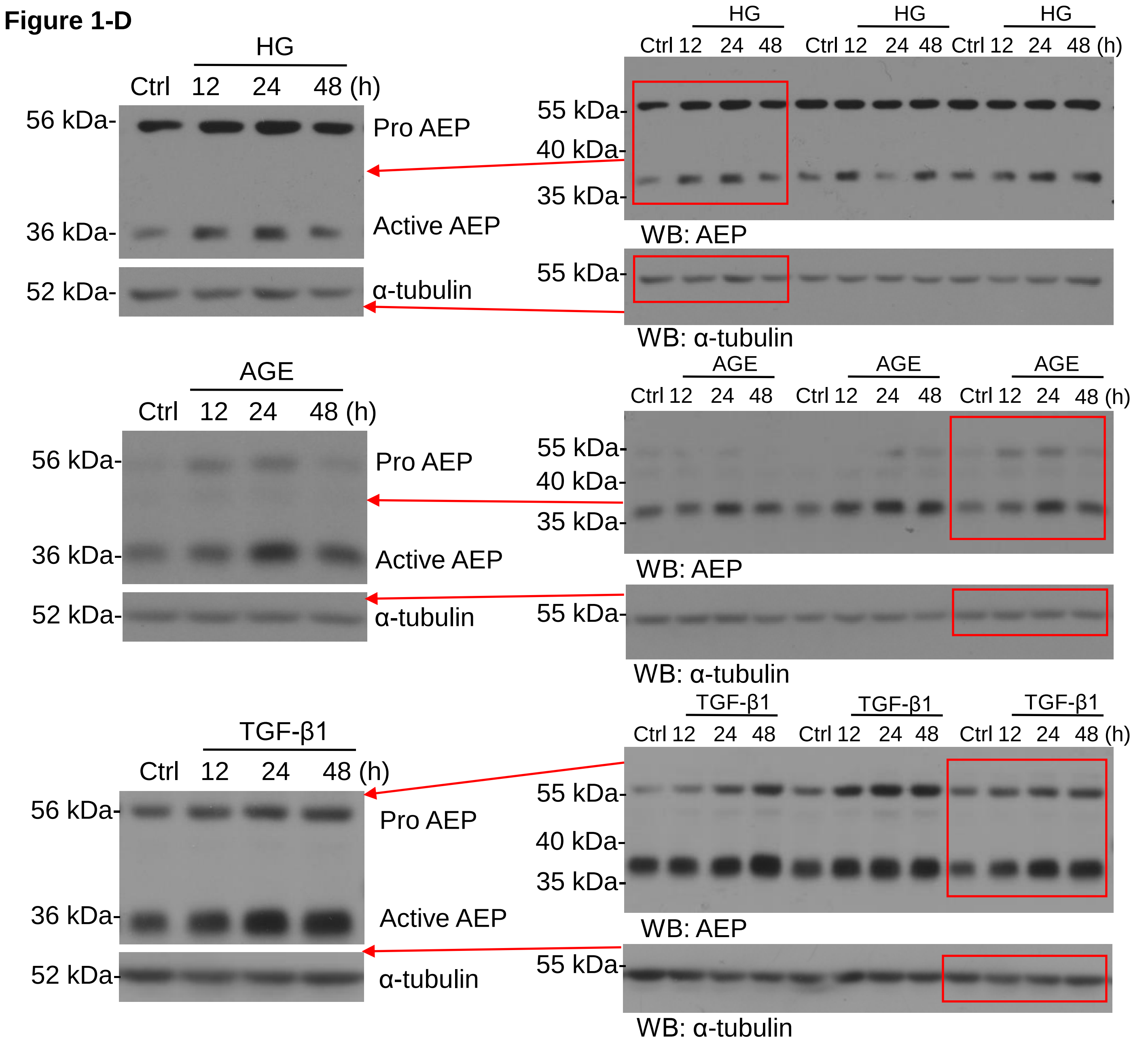

Figure 2-A

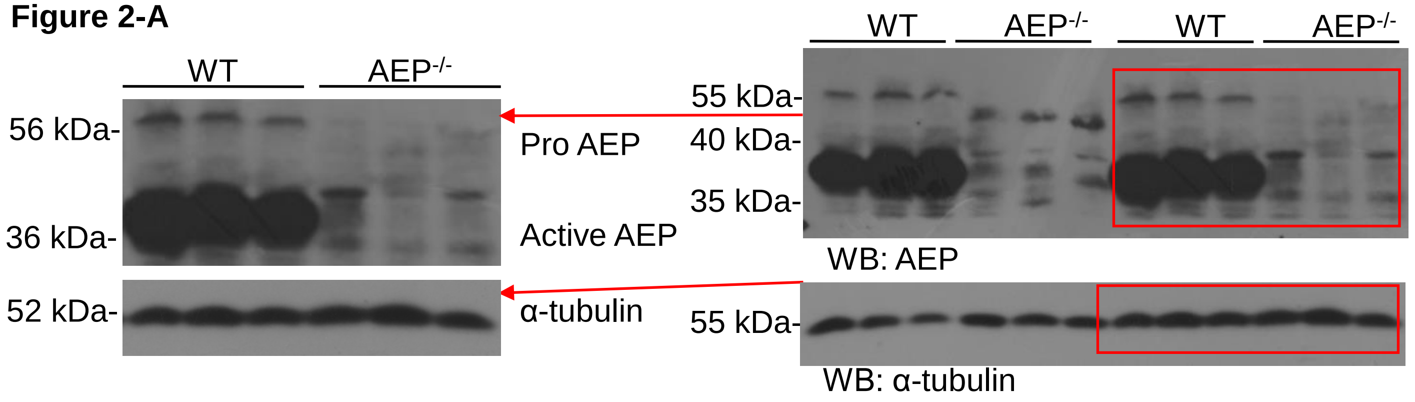

Figure 4-C

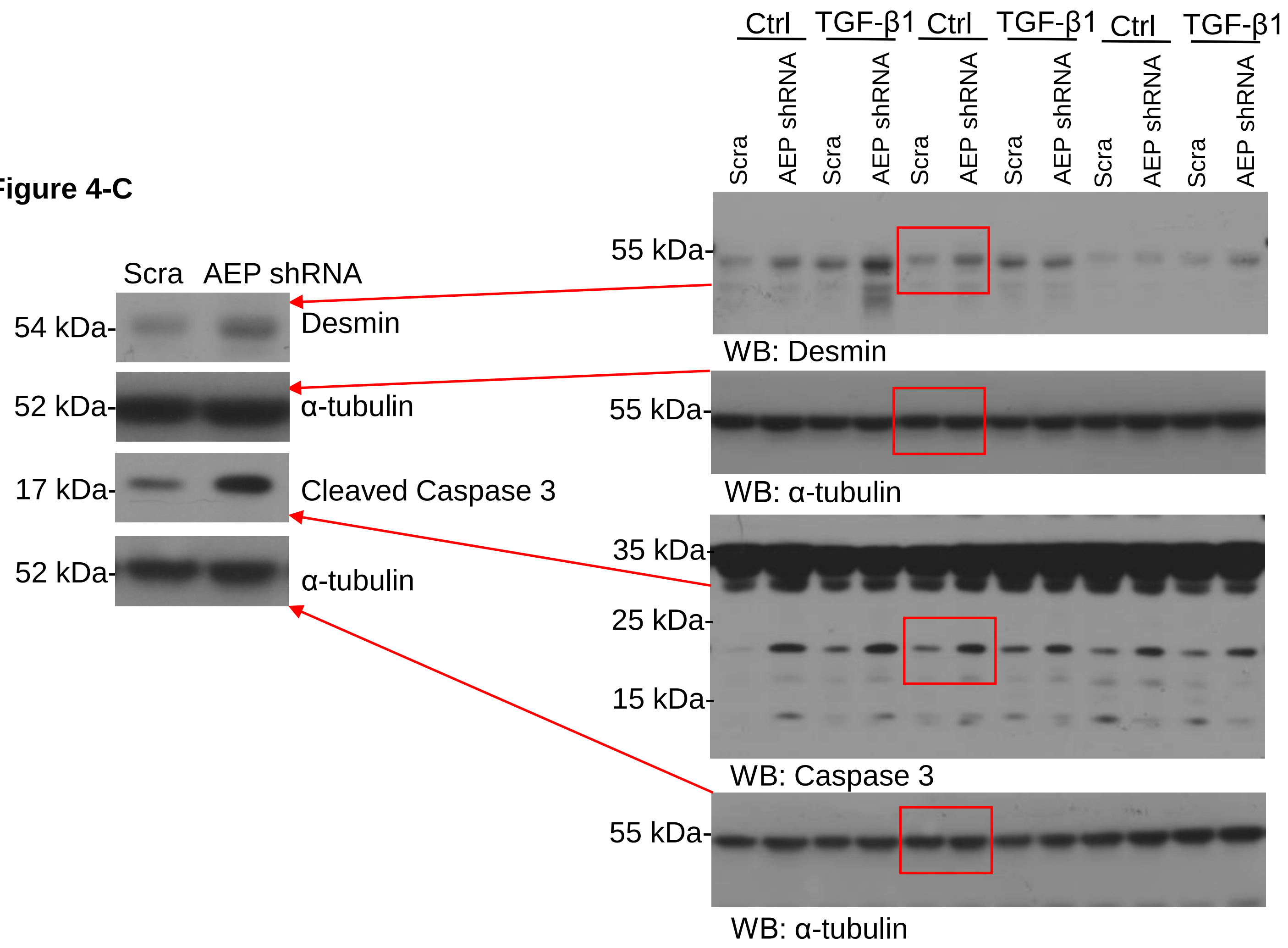

Figure 4-E

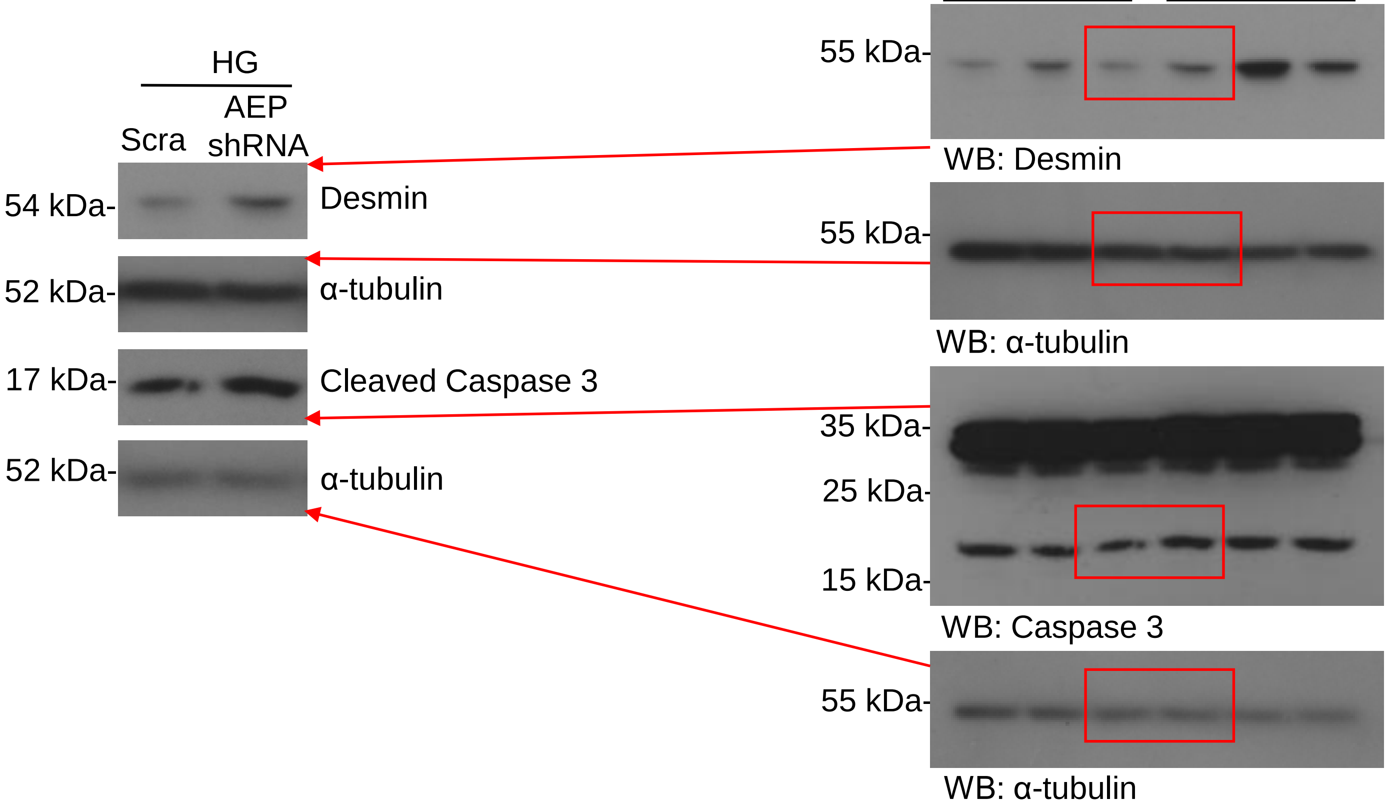

Figure 4-I

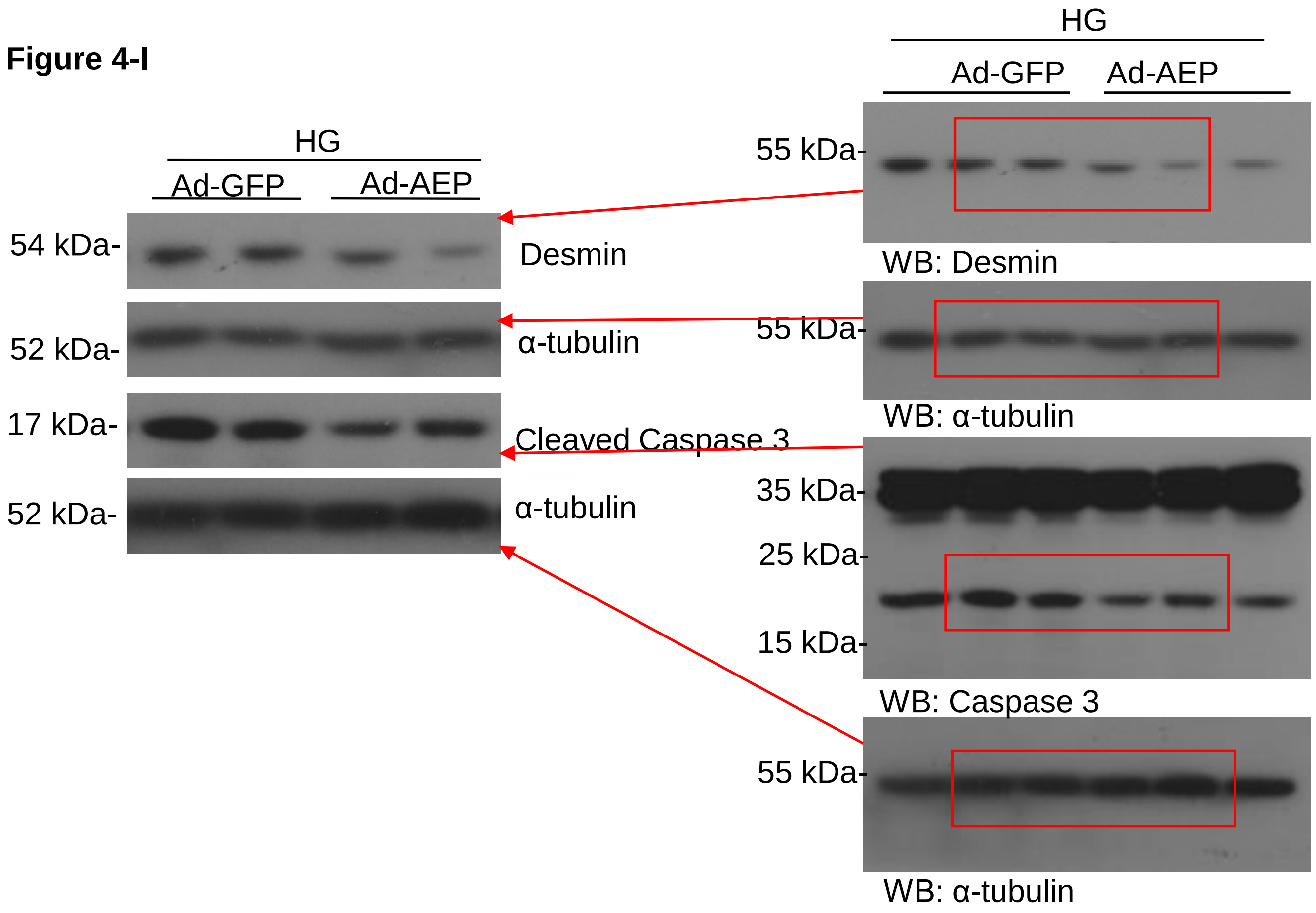

Figure 5-A

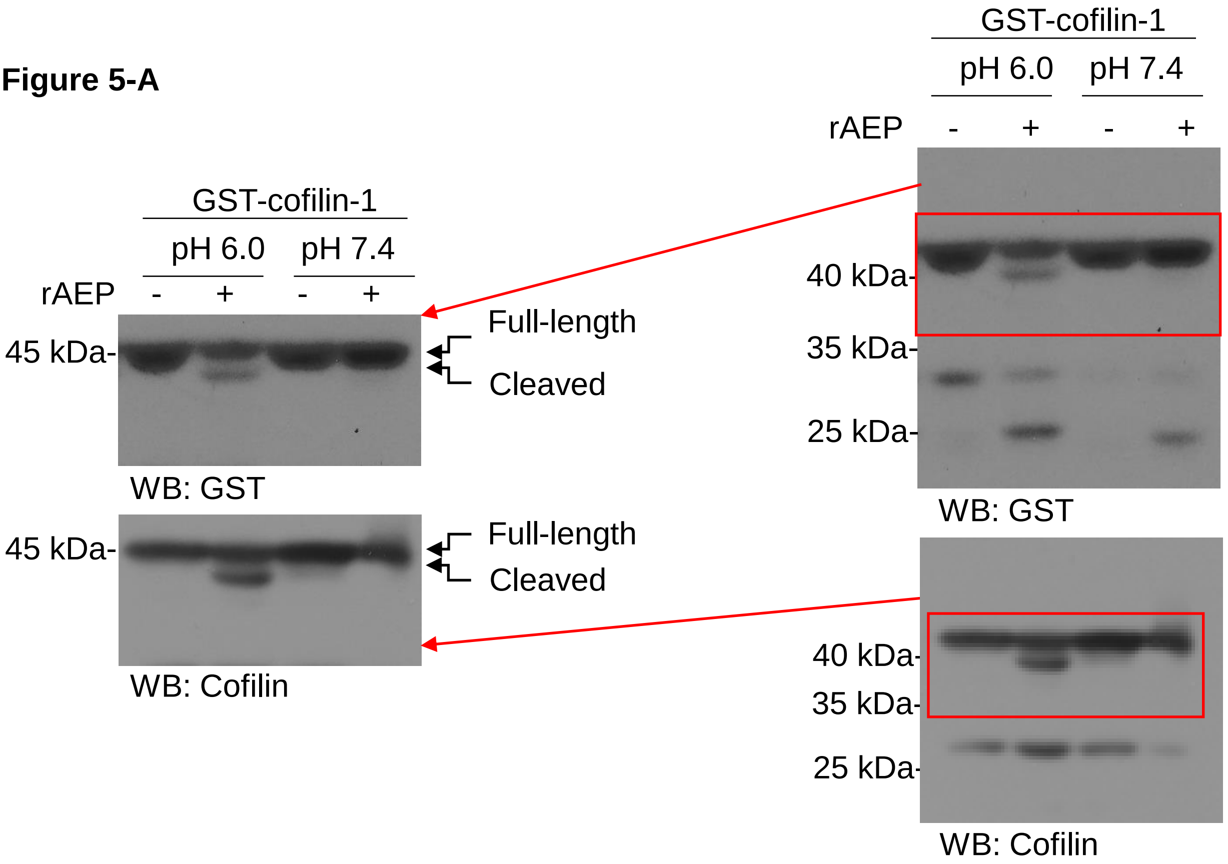

Figure 5-B

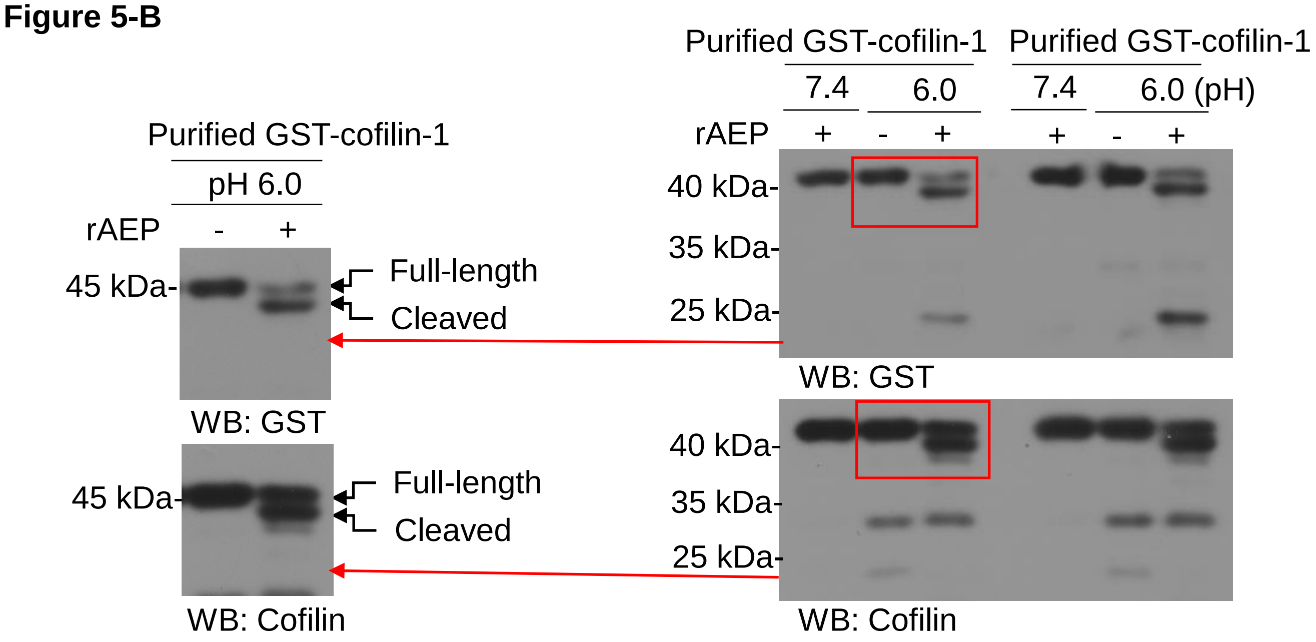

Figure 5-C

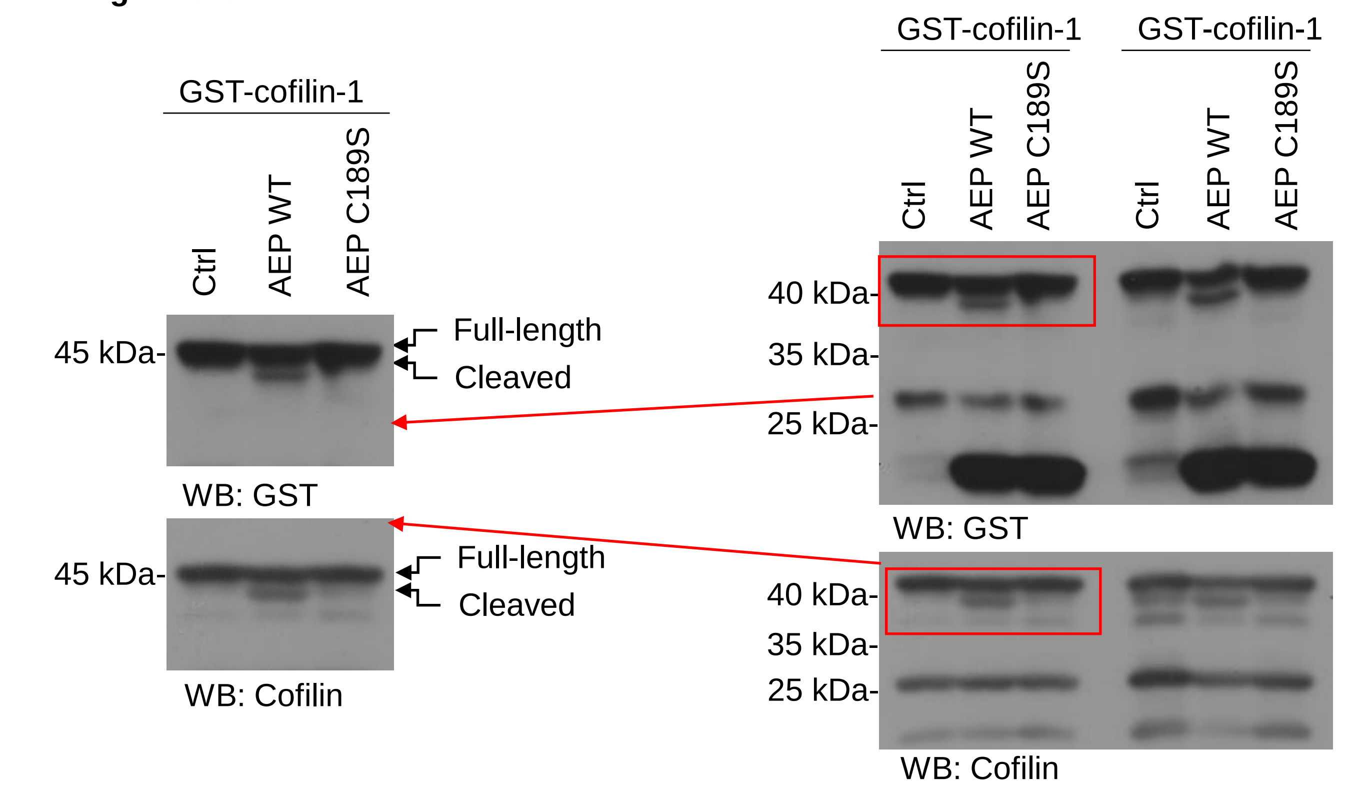

Figure 5-D

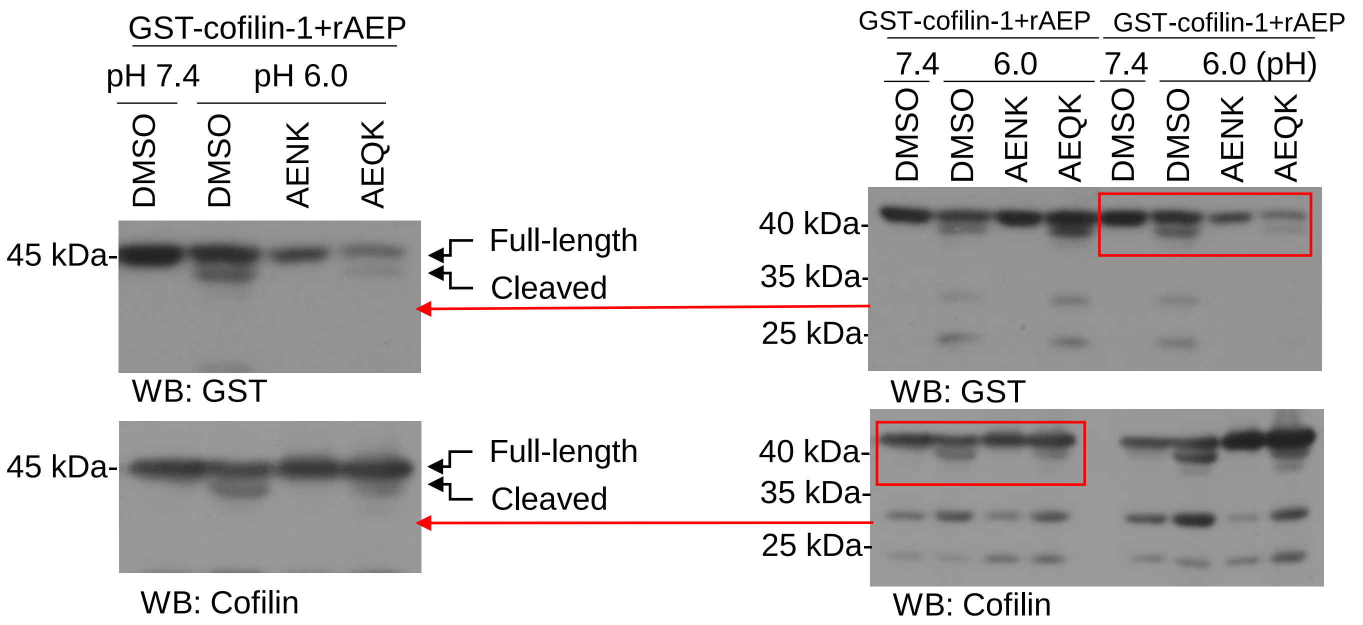

Figure 6-C

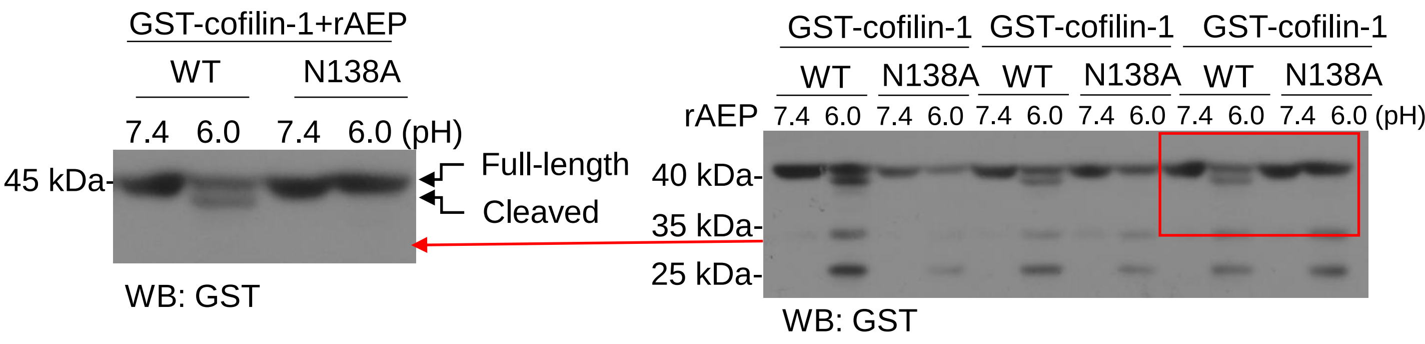

Figure 6-D

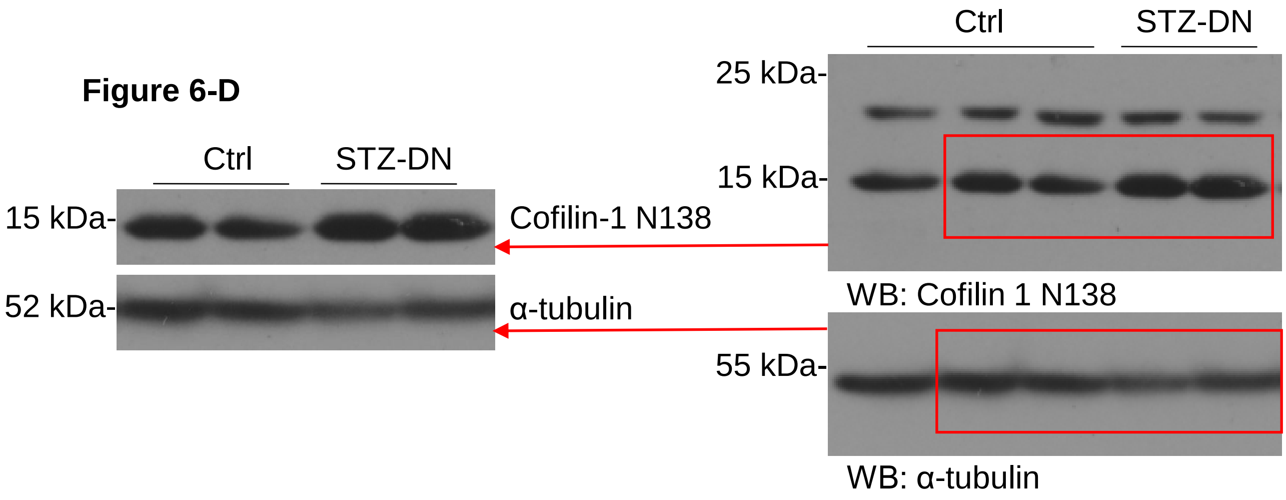

Figure 6-H

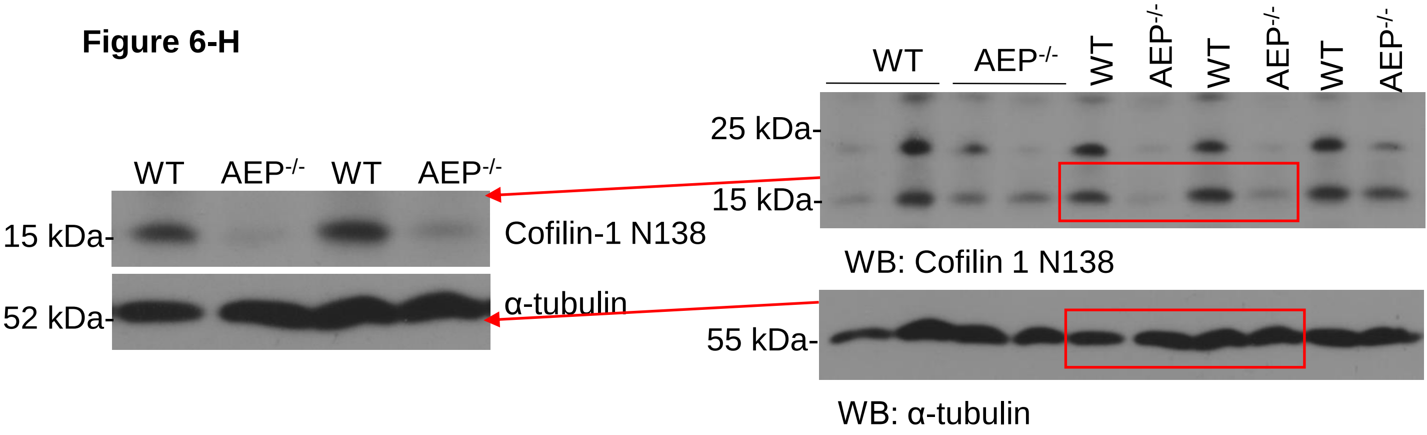

Figure 7-A

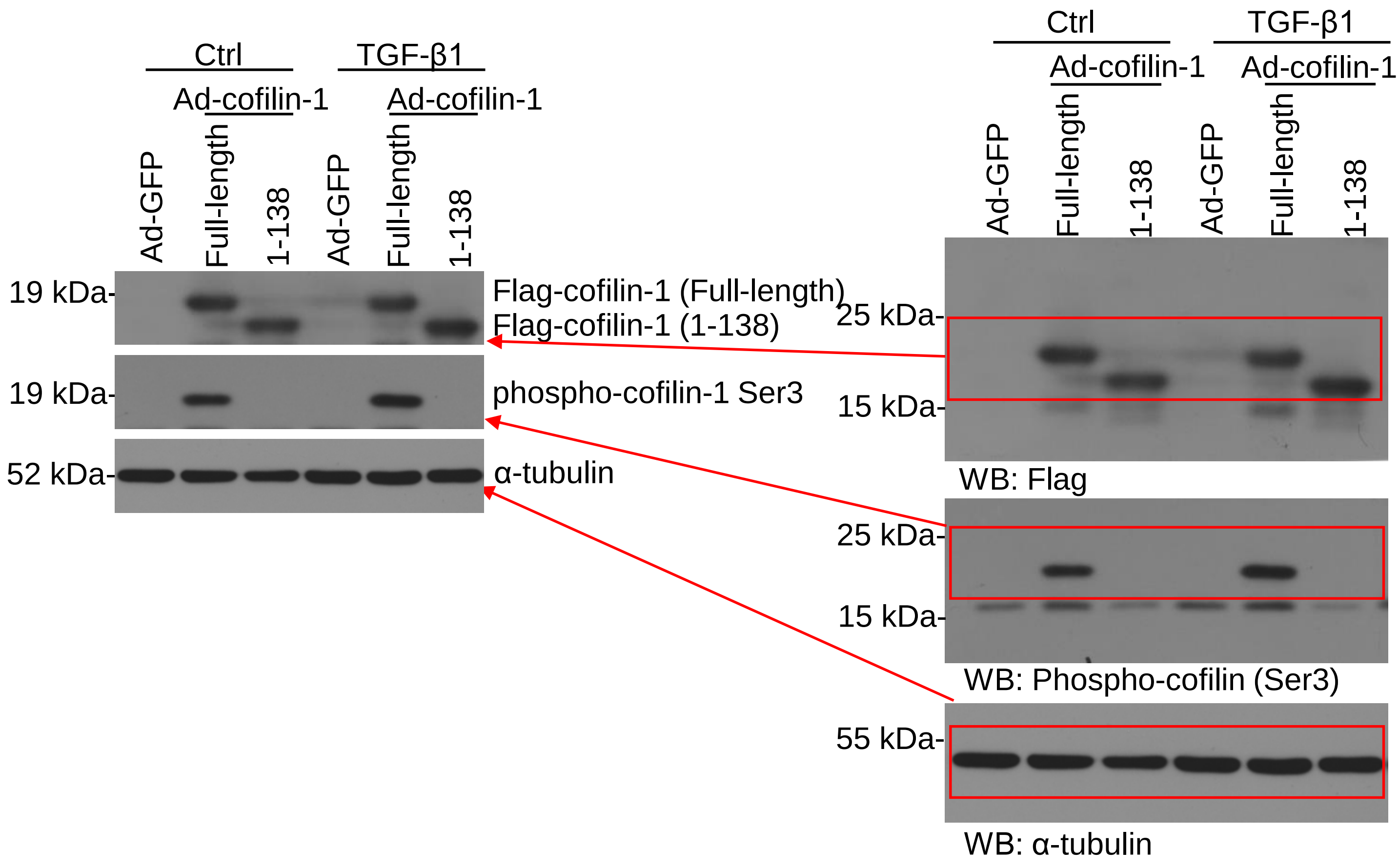

Figure 7-D

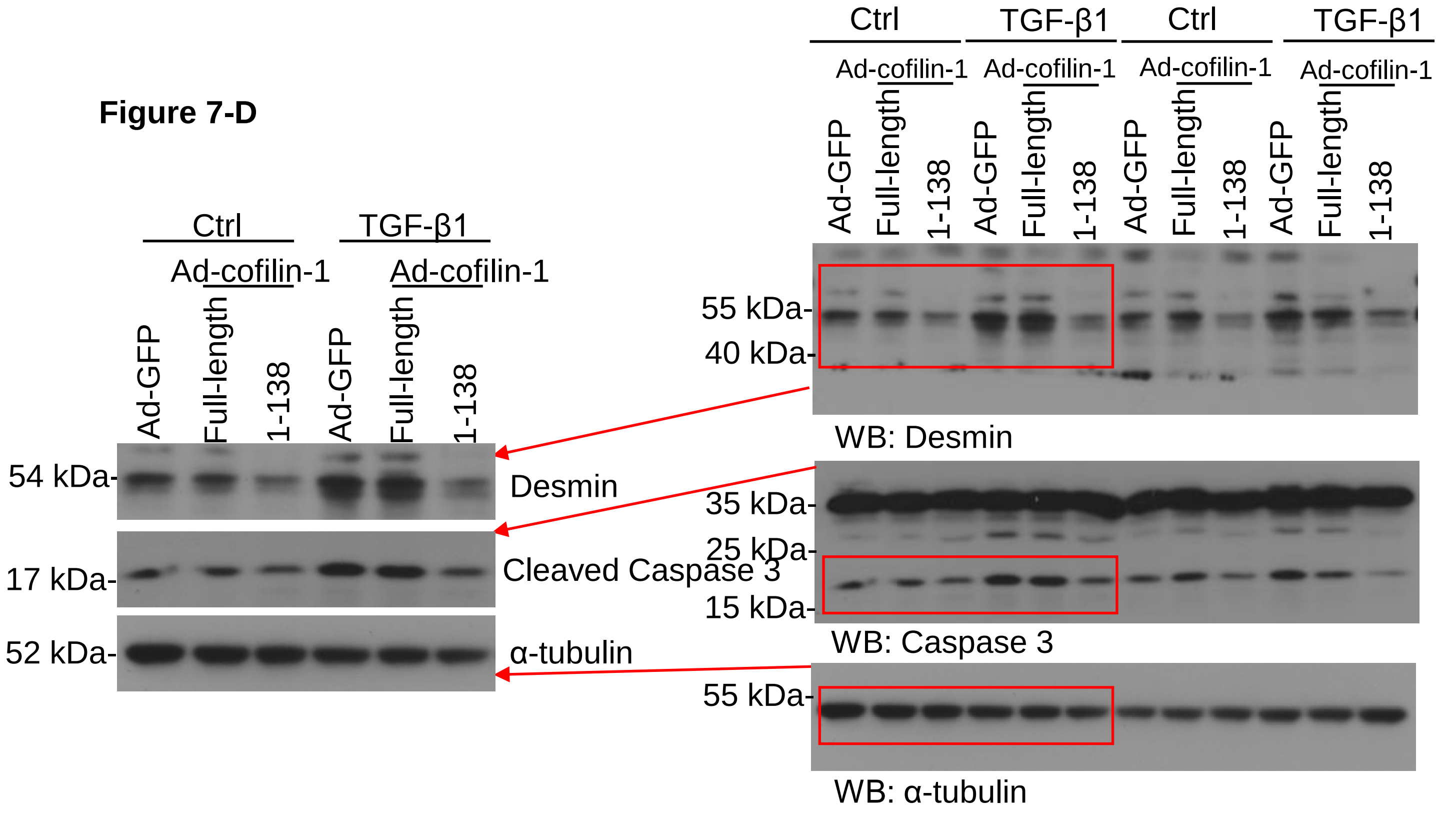

Supplemental Figure 1-A

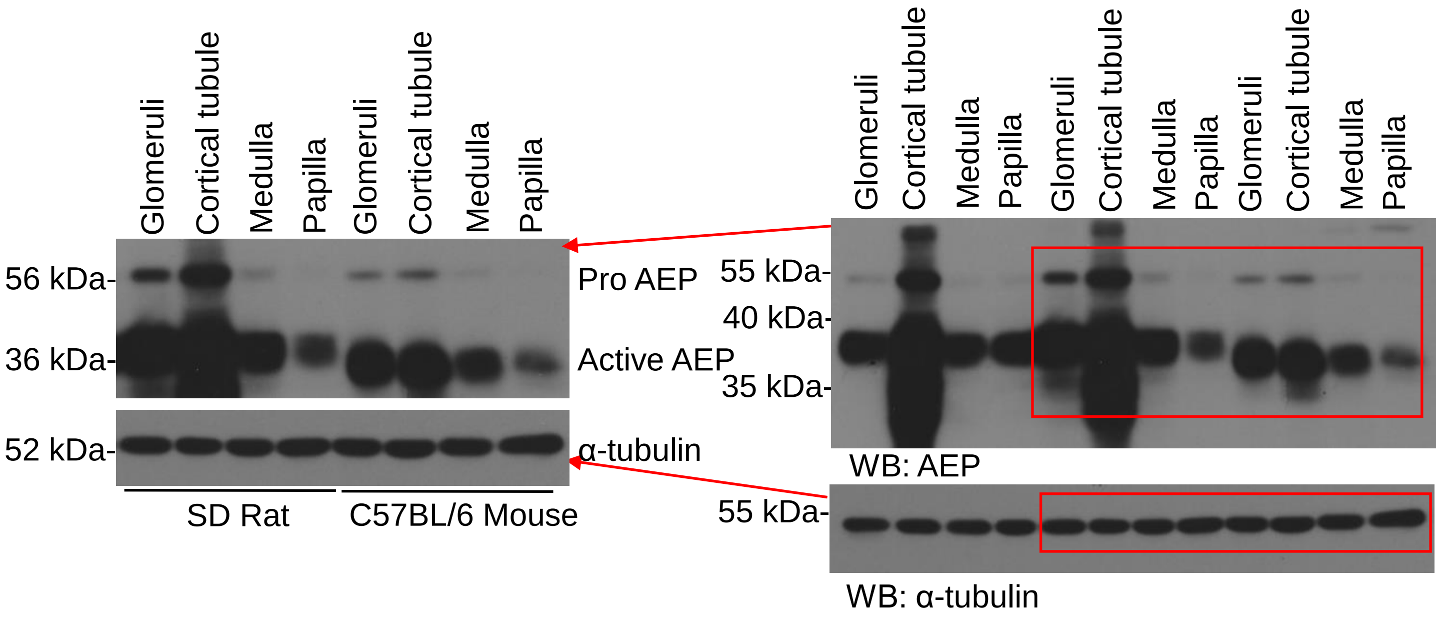

Supplemental Figure 1-C

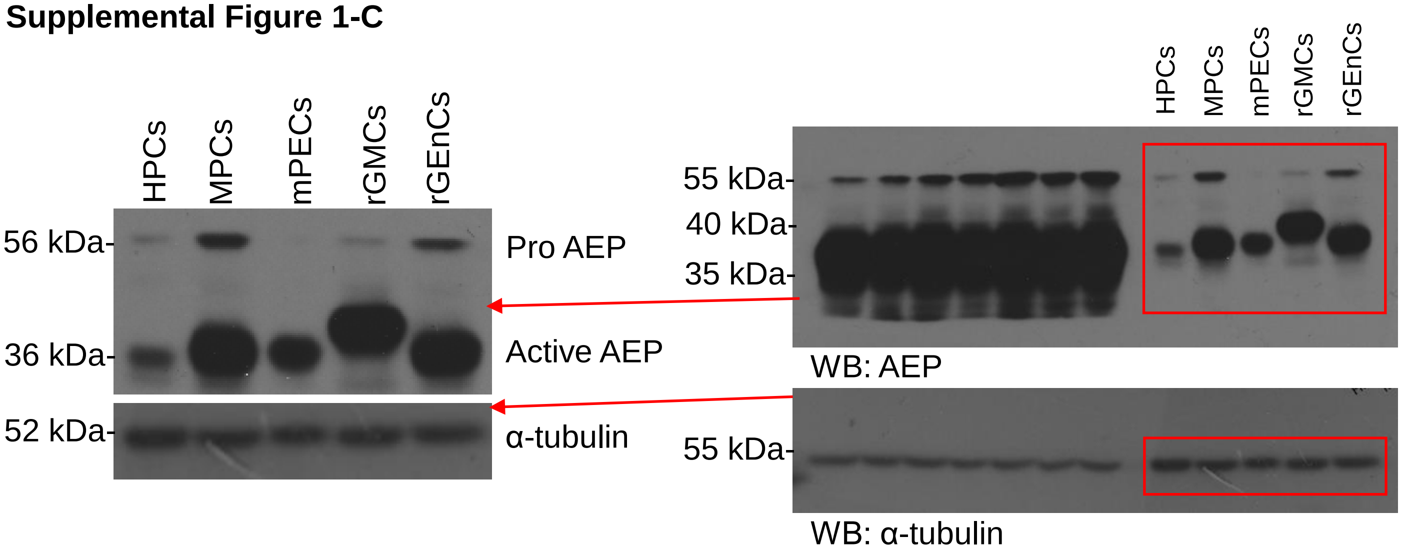

Supplemental Figure 5-A

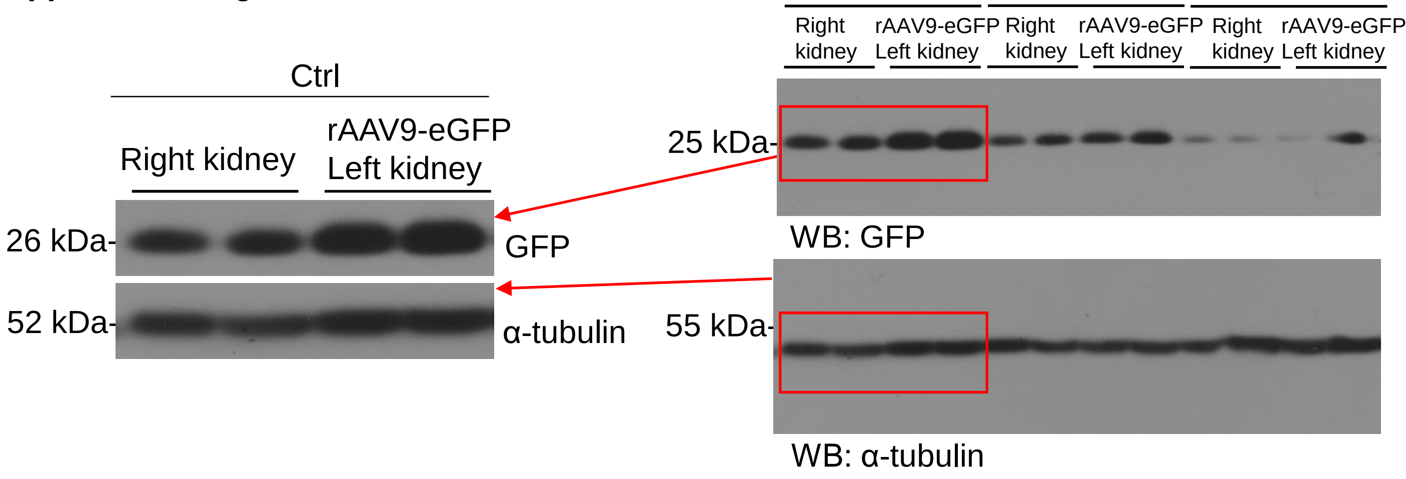

Supplemental Figure 5-C

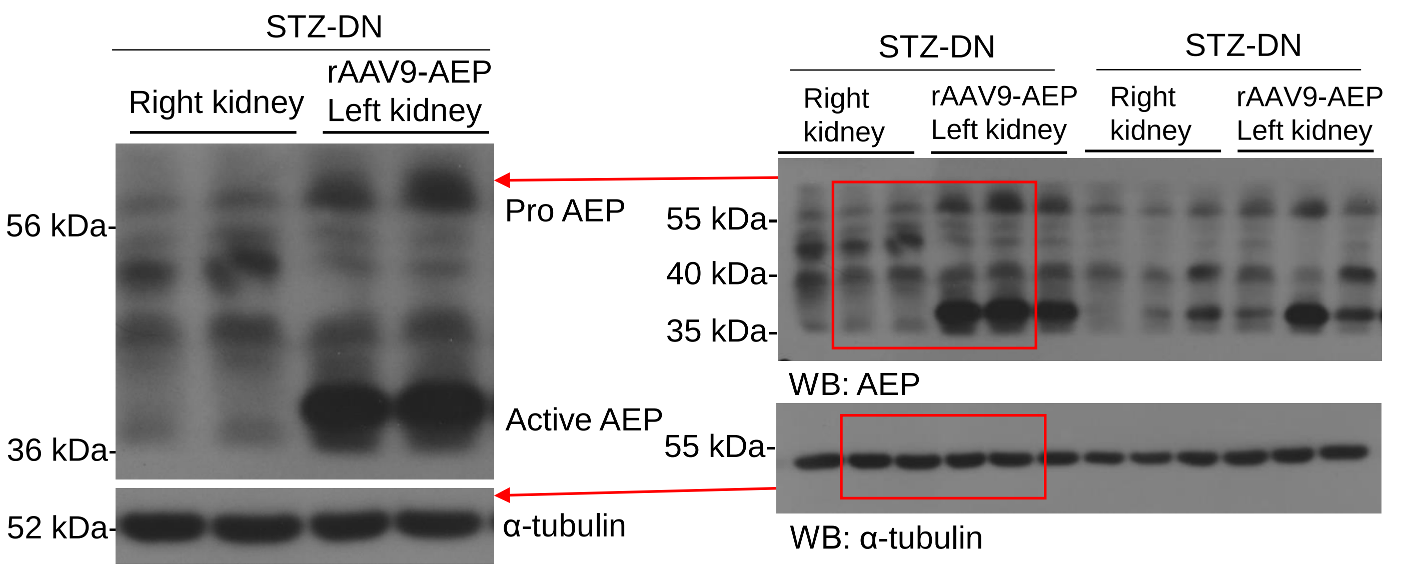

Supplemental Figure 6-A

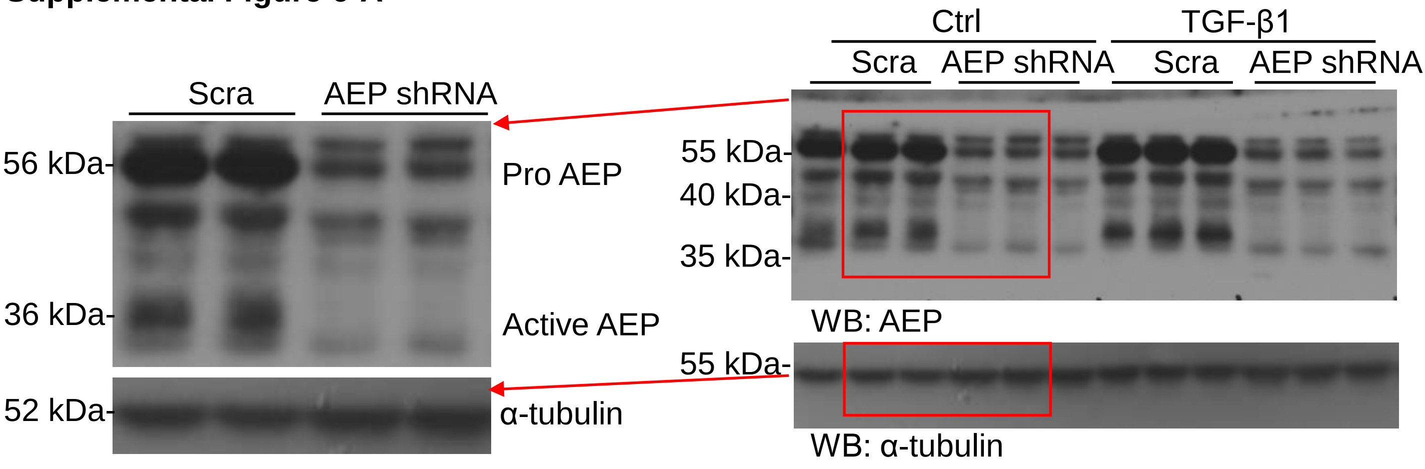

Supplemental Figure 6-C

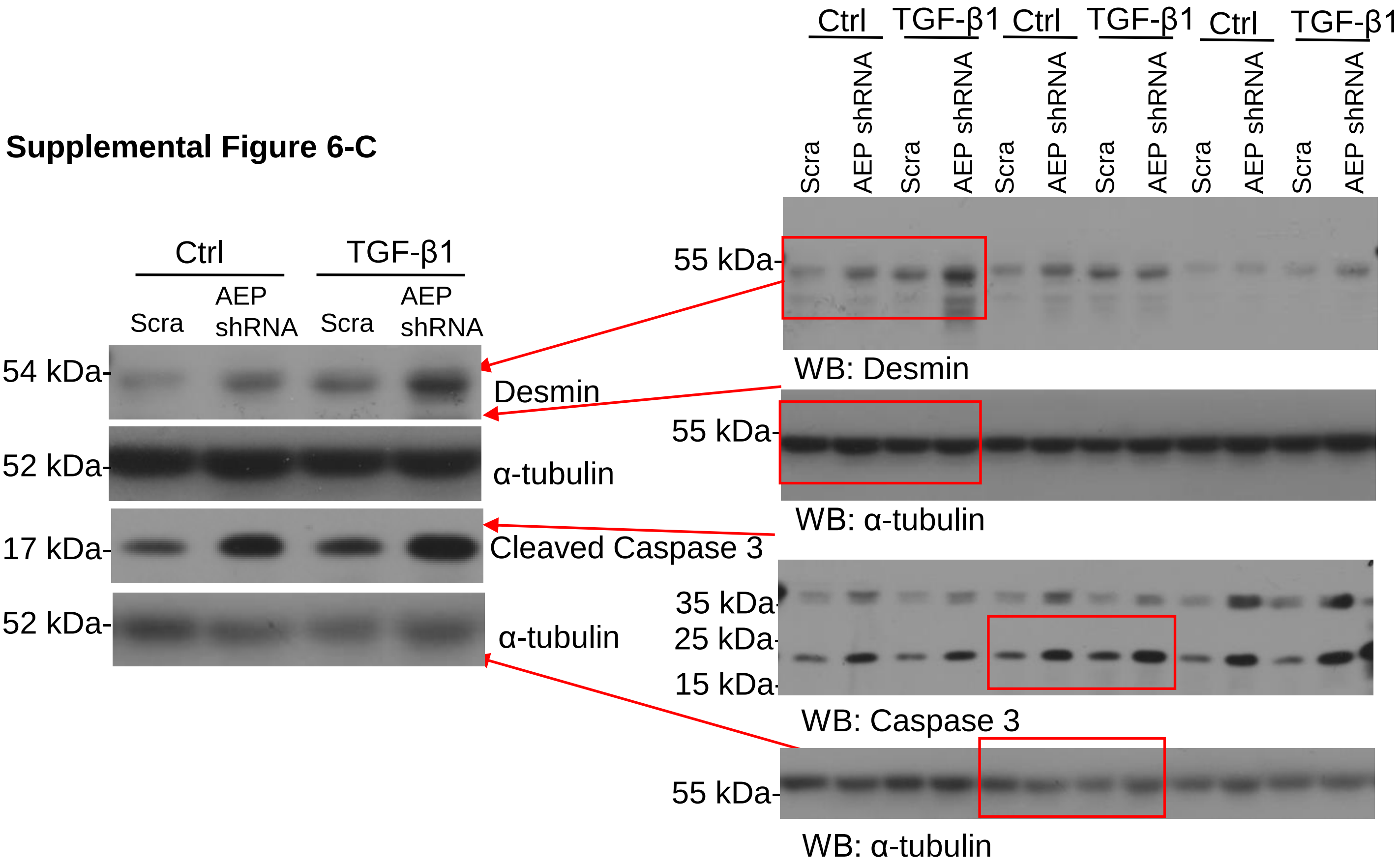

Supplemental Figure 7-A

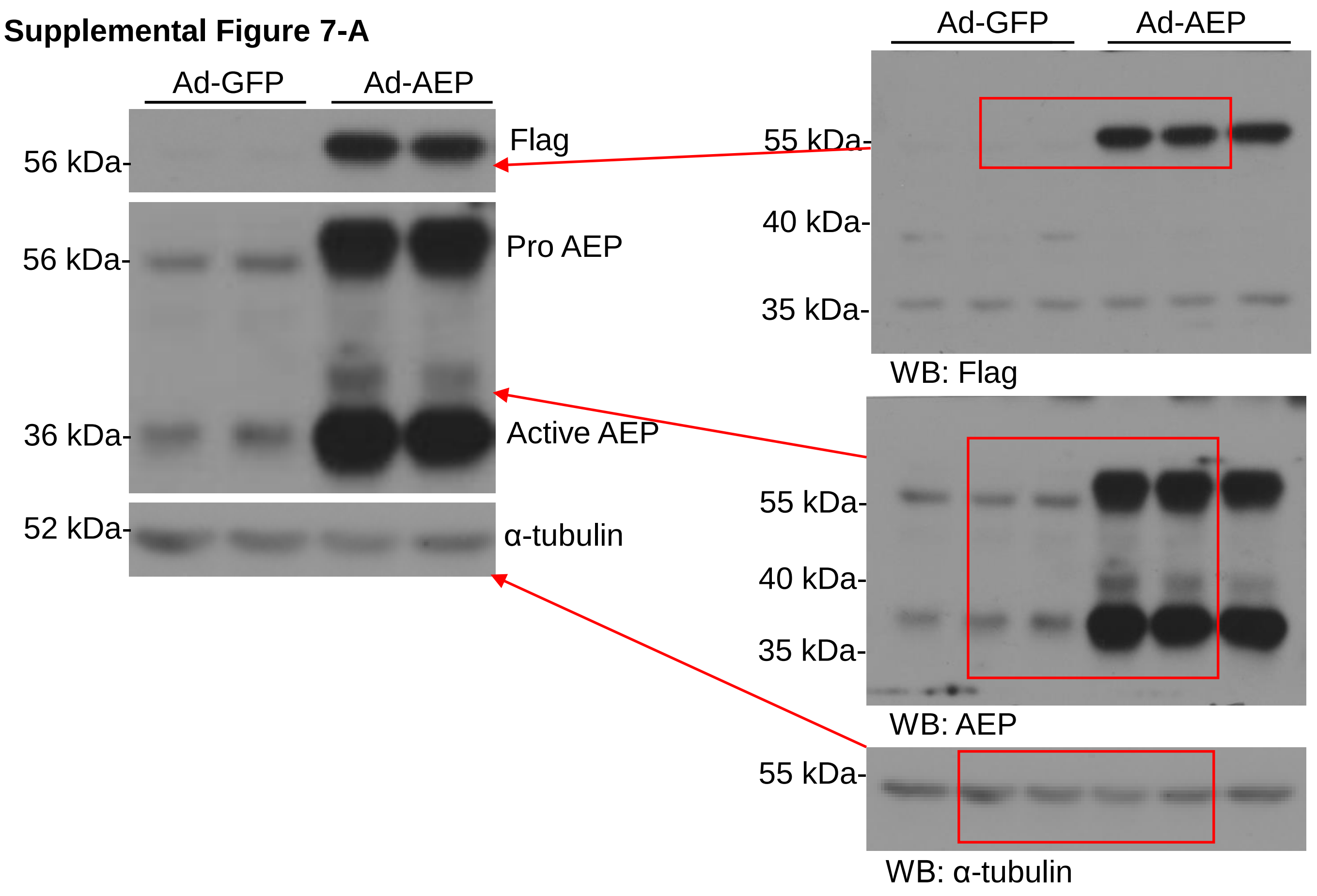

Supplemental Figure 7-E

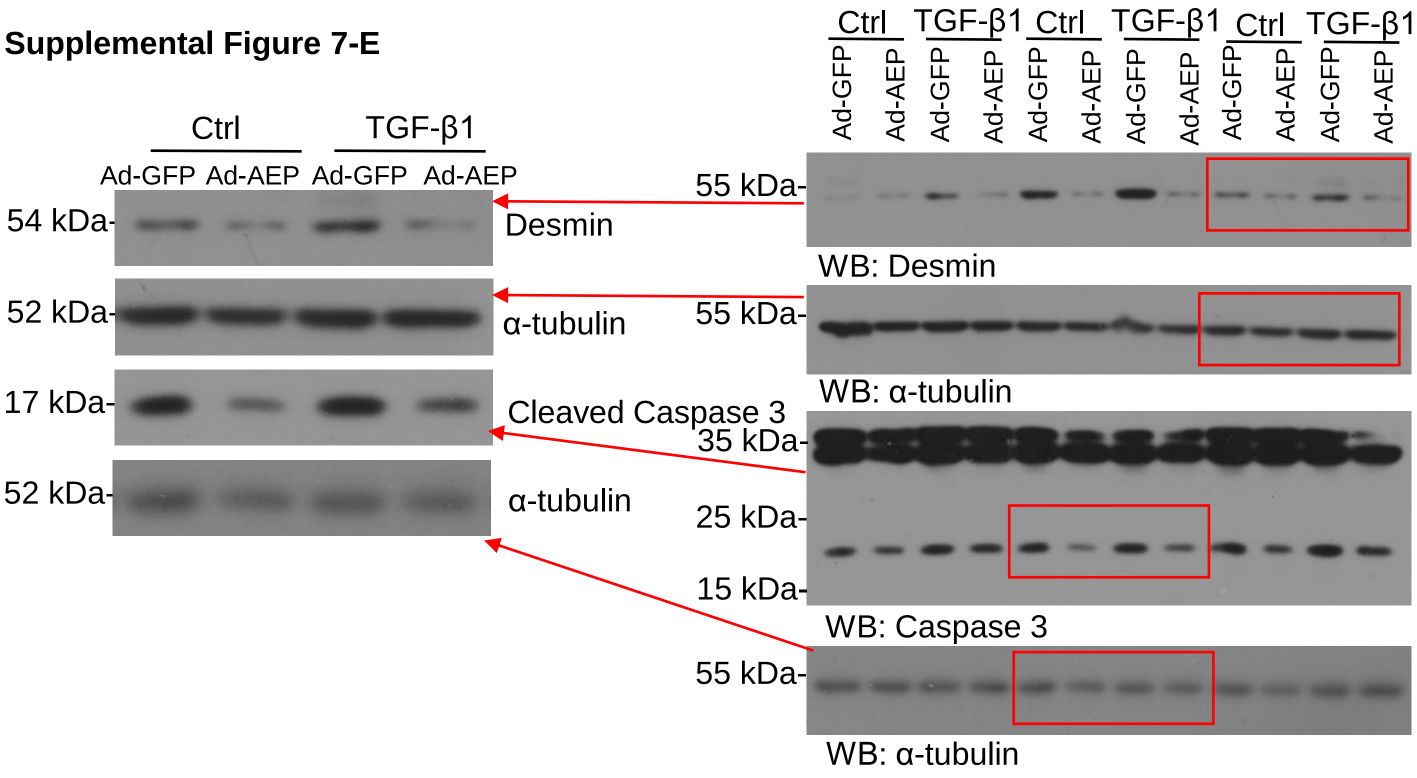

Supplement: Supplementary file 2 — Western blot gel images [file 41419_2022_4621_MOESM2_ESM.pdf]
